# Chromosomal clustering of a human transcriptome reveals regulatory background

**DOI:** 10.1186/1471-2105-6-230

**Published:** 2005-09-19

**Authors:** Jan H Vogel, Anja von Heydebreck, Antje Purmann, Silke Sperling

**Affiliations:** 1 Cardiovascular Genetics Group, Department of Vertebrate Genomics, Max Planck Institute for Molecular Genetics, Ihnestrasse 73, 14195 Berlin, Germany; 2 Department of Computational Molecular Biology, Max Planck Institute for Molecular Genetics, Ihnestrasse 73, 14195 Berlin, Germany

## Abstract

**Background:**

There has been much evidence recently for a link between transcriptional regulation and chromosomal gene order, but the relationship between genomic organization, regulation and gene function in higher eukaryotes remains to be precisely defined.

**Results:**

Here, we present evidence for organization of a large proportion of a human transcriptome into gene clusters throughout the genome, which are partly regulated by the same transcription factors, share biological functions and are characterized by non-housekeeping genes. This analysis was based on the cardiac transcriptome identified by our genome-wide array analysis of 55 human heart samples. We found 37% of these genes to be arranged mainly in adjacent pairs or triplets. A significant number of pairs of adjacent genes are putatively regulated by common transcription factors (p = 0.02). Furthermore, these gene pairs share a significant number of GO functional classification terms. We show that the human cardiac transcriptome is organized into many small clusters across the whole genome, rather than being concentrated in a few larger clusters.

**Conclusion:**

Our findings suggest that genes expressed in concert are organized in a linear arrangement for coordinated regulation. Determining the relationship between gene arrangement, regulation and nuclear organization as well as gene function will have broad biological implications.

## Background

To understand the global regulatory network underlying specific transcriptomes, several distinct aspects have to be considered [1,2]; (A) the genomic organization of those transcripts [3], (B) their regulation by general and specific transcription factors, (C) the influence of epigenetic effects such as e.g. histone modifications [4], (D) the local environment [5,6], and (D) the functional role of the transcripts as well as their protein products as nodes of the network. In the present report, we show for the first time for a human transcriptome that there is a relationship between the genomic organization, transcriptional regulation and functional role.

It has long been known that transcriptional regulation is related to chromosomal gene order; prokaryotic operons are the best known example [7]. For lower eukaryotes coexpressed adjacent genes were first described in Saccharomyces cerevisiae [8,9]. For a part of those gene pairs, a common transcriptional activation was proposed through a shared upstream activating sequence, which occurs in the promoter region of one of the two genes. Furthermore, correlated triplets, but not quadruples, were found to occur more often than expected in yeast. Reports for Caenorhabditis elegans [10], Drosophila melanogaster [11,12], Homo sapiens and Mus musculus [13-15] showed coexpression of co-localized genes in higher eukaryotes, and reports of particular gene cluster such as the human β-globin locus [16], the interleukin-13 gene locus [17] and others [18-20] indicate the association to regulated chromatin domains. However, on a global scale only few insights into the molecular mechanisms of the transcriptional regulation of clustered genes have been gathered so far, and data about small clusters of adjacent genes have only been partially analyzed. Beside the finding of housekeeping gene clusters throughout the human genome [14], no evidence for a functional correlation of clustered genes had been shown. However, coexpressed genes in general (regardless of their localization) appear to function in similar biological processes [21].

In this paper we describe the chromosomal co-localization in adjacent pairs of a large proportion of the human transcriptome in heart in the context of their expression dynamics, their transcriptional regulation and their function in shared biological processes. We examined the cardiac transcriptome using data from our previous genome-wide array analysis [22] and found profound evidence for a significant clustering of more than 37% of those genes located mainly in pairs or triplets. A significant proportion of these clustered genes have common putative transcription factor binding sites within their promoter regions and share common biological functions.

## Results

**Figure 1:**
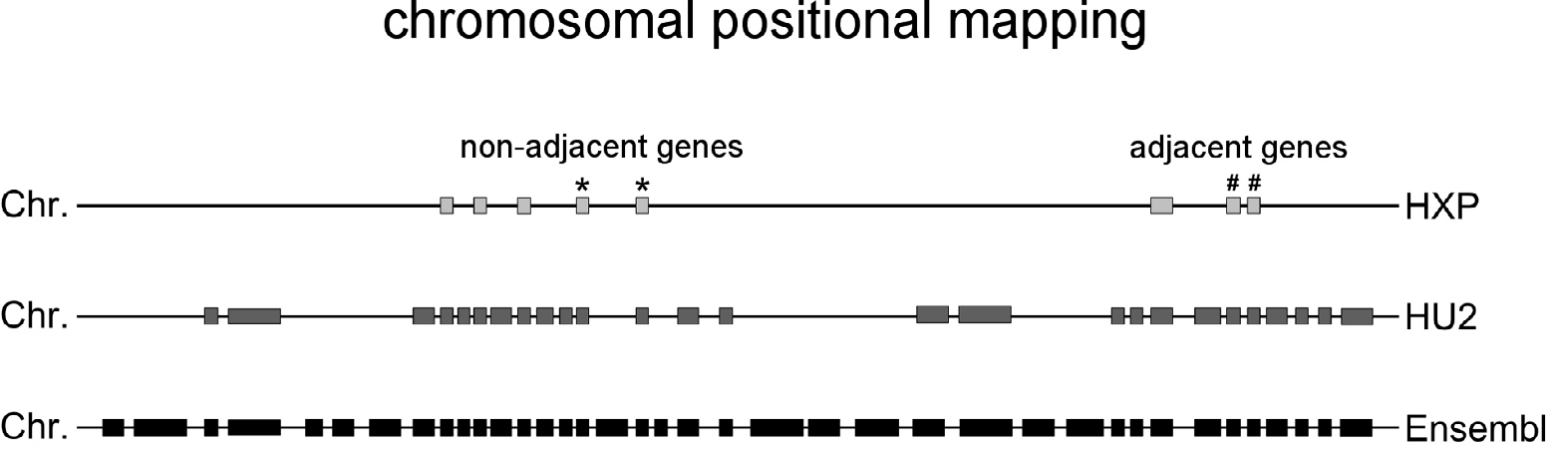
**Gene localization based on Ensembl, HU2 and HXP**. Genes marked with * illustrate an adjacent gene pair in reference to HU2 and HXP, but not referring to the Ensembl gene set (131 cases). Therefore, only genes like those marked with # are noted as clusters throughout.

To characterize genomic organization of the cardiac transcriptome, we investigated a set of 3.172 heart-expressed genes (HXP) identified in our previous study that represent the cardiac transcriptome based on the analysis of 55 human heart samples [22]. This gene set reflects all genes continuously transcribed in the analyzed heart samples. Thus, we focused on the information whether or not a gene and herewith a particular genomic region is transcribed at all and considered expressed neighboring genes as coexpressed gene clusters regardless of the expression levels of individual genes. We assigned the position of this HXP set with regard to the whole human genome as represented by Ensembl compared to the HU2 gene set represented on the arrays. In order to reflect the actual adjacency of genes on the chromosomes, we defined gene neighbors according to their Ensembl annotation, rather than using the HU2 gene set as a basis (Fig. 1).

### Chromosomal distribution and gene clusters

**Figure 2:**
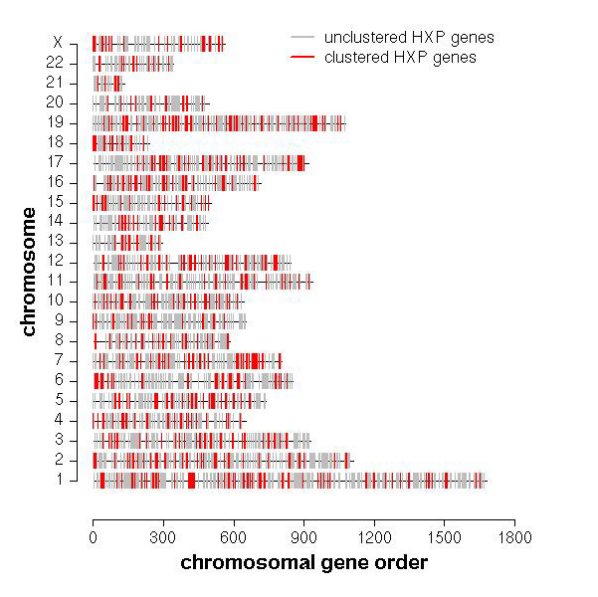
**Genomic organization of the overall and clustered heart-expressed genes**. The chromosomal gene order of clustered genes is represented in red, whereas the non-clustered genes are shown in gray, both appear to distribute without notable prevalence among the chromosomes.

First, we analyzed the overall chromosomal distribution of HXP and observed no overrepresentation of HXP on specific chromosomes (Chi-Square-Test, p = 0.2). On average each chromosome contained a proportion of 18% HXP genes out of the overall analyzed dataset. Upon closer analysis of the distinct localization of HXP genes, we observed small groups of physically adjacent genes along the chromosomes throughout the genome. In Figure 2 the chromosomal distribution of the overall HXP genes and neighboring coexpressed HXP genes is represented. We calculated the number of gene clusters made up of two to five physically adjacent HXP genes and measured the statistical significance of this local clustering by comparing the numbers of HXP gene clusters with a random distribution obtained by 100,000 permutations of HU2. We observed a significantly higher number of adjacent gene pairs (881 genes, p = 0.01) and gene triplets (307 genes, p = 0.02) in HXP than would be expected for a random distribution, whereas the number of quadruples and quintuples did not differ significantly (Table 1). In total, we found 1,179 HXP genes to be locally clustered. Further, we analyzed whether there was any prevalence regarding gene orientation within these clusters compared to a random distribution obtained by 10,000 permutations. Besides an enrichment of co-oriented gene pairs within clusters of size ≥ 2 (p = 0.03), we observed no bias in the numbers of anti-oriented gene pairs in clusters ≥ 2 (p = 0.2) as well as for co- and anti-oriented gene triplets in clusters ≥ 3 (p = 0.3 and p = 0.6, respectively).

**Table 1:** Numbers of detected heart-expressed gene clusters of different sizes.

Cluster-size	Number of gene cluster	ENSG	p-value	Z-score
2	480	881	0.01	3.89
3	79	207	0.02	3.24
4	15	57	0.05	2.68
5	1	5	0.7	0.07

ENSG represents the number of unique Ensembl genes in all clusters of the given size. The p-values and Z-scores result from a comparison with a random distribution obtained by 100,000 permutations of the HU2 dataset.

### Gene clusters and housekeeping functionality

**Figure 3:**
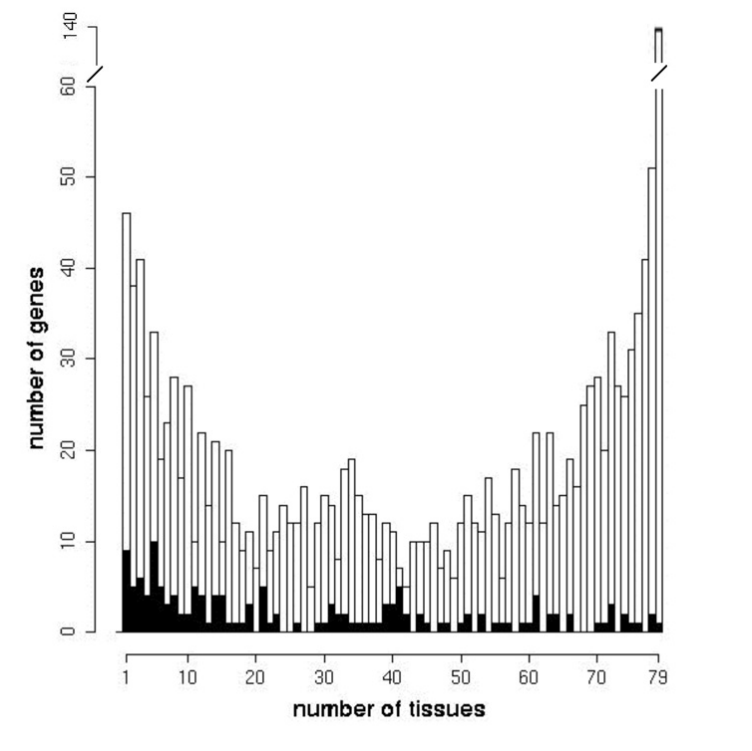
**Tissue expression of overall and clustered HXP genes**. The numbers of tissue expression of clustered heart-expressed genes are presented filled black. Of the 79 analyzed tissues, the expression profile of clustered genes shows a broad panel of tissue expression but only very few clustered genes are expressed in the majority of tissues. Thus we concluded that clustered coexpressed genes are mainly non-housekeeping genes.

Previously, it had been suggested that housekeeping genes are arranged in clusters along the genome [14]. Therefore, we assessed the tissue expression of our HXP gene set and the subset of coexpressed adjacent genes in 79 human tissues, for which the expression information of protein-coding genes had been recorded by the GNF Symatlas [15] (Fig. 3). We observed a slightly bimodal distribution for the overall HXP gene set, with a major peak corresponding to expression in 79 tissues. This distribution differed from the one observed for coexpressed adjacent genes. Here, the majority of genes showed an expression in a distinct number of tissues, not reflecting a housekeeping-like expression profile.

### Transcriptional regulation of gene clusters

We extended our analysis to determine to what extent these gene clusters are regulated by common transcription factors. For this purpose, we identified the putative transcription factor binding sites (TFBS) for the HXP set using the CORG database [23]. CORG is based on TFBS in non-coding regions conserved between human and mouse (see Methods), and enabled us to identify binding sites of 276 distinct binding site models in the promoter regions of a total of 1,777 HXP genes. For the remaining HXP genes no conserved non-coding region could be identified.

Taking into account that several models can represent binding sites of one transcription factor, the identified TFBS pertained to only 216 distinct transcription factors. Within the HXP set with assigned TFBS, 501 genes belonged to chromosomally clustered gene pairs and for 171 pairs, binding sites for both genes were predicted [see Additional file 1]. We observed the largest number of common predicted transcription factors for the antisense-sense gene pair consisting of the *HOOK2 *protein and transcription factor *JUNB*, which share binding sites for 64 transcription factors. The mean number of observed common transcription factors predicted to regulate adjacent genes was 4.3. There were 23 genes in the HXP set with binding sites for more than 75 different transcription factors, which we consider to be outliers.

**Figure 4:**
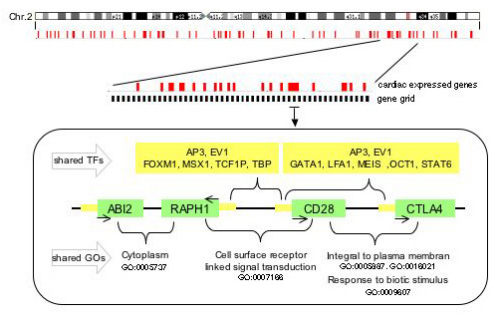
**Example of four adjacent coexpressed genes**. Shown is the gene cluster distribution on human chromosome 2 with an example of four adjacent genes regulated in part by common transcription factors and sharing gene ontology categories. Coexpressed gene clusters are shown in red. Each coexpressed gene pair ABI2 – RAPH1; RAPH1 – CD28 and CD28 – CTLA4 shares GO terms as indicated. Genes of the triplet RAPH1 – CD28 – CTLA4 have transcription factor binding sites for AP3 and EV1 in common. Furthermore, each gene pair RAPH1 – CD28 and CD28 – CTLA4 shares additional TFBS. Genes are marked in green, arrows indicate the strand orientation, promotor regions and transcription factors are colored in yellow.

Finally, we tested the significance of the observed number of gene pairs potentially regulated by at least one common transcription factor by comparing this number to the numbers of gene pairs with commonly assigned transcription factors seen in 10,000 random permutations of HXP genes. Regardless of whether the calculation was based on the number of distinct TFBS or distinct transcription factors, the common transcriptional regulation was significantly greater within the observed gene clusters than expected from the random distribution (p = 0.02 and p = 0.03, respectively). This significance was not influenced by genes defined as outliers with regard to their large number of common TFBS. Furthermore, the identified common transcription factors belonged to a broad panel of transcription factors classes without prevalence of particular families. Taking the gene orientation into account again, we found a homogenous distribution of strand orientation within observed gene pairs regulated by common transcription factors. An example of a gene cluster regulated by common transcription factors is given in Figure 4.

### Functional relationship of gene clusters

Further, we analyzed to what extent heart-expressed genes organized as adjacent pairs are involved in the same biological process using the Gene Ontology (GO) database as a source of annotations of biological functions. Naturally, our analysis was limited to those genes annotated with GO terms that were around 60% of all HXP genes (1,921 genes) including those located in 176 gene clusters. A total of 2,158 different GO terms could be assigned to the overall HXP set with 1,241 GO terms mapping to genes located in clusters. To focus on non-generic functional annotations, we calculated the number of GO terms shared within pairs of adjacent HXP genes for the 5^th ^and 6^th ^level of granularity of the GO hierarchy and compared these to a random distribution generated from HXP genes to account for the tissue-specificity of the analyzed dataset. We observed a significant enrichment of shared GO terms within pairs of adjacent genes for both levels of granularity (p << 0.0001 for each). Only eight of the 96 and 45 pairs sharing the 5^th ^and 6^th ^level of functional classification are conspicuous in terms of having originated by gene duplications [see Additional file 2] (for an example see Fig. 4).

### Expression pattern of adjacent genes

Finally, we attempted to determine whether genes located close to each other show a correlation of their expression levels within our previously analyzed patient cohort, which consisted of 5 cardiac phenotypes with characteristic expression profiles [22]. First, we built correlation maps for visual inspection of the overall HXP gene set. To construct such matrices, we sorted the HXP genes of each chromosome according to their arrangement on the chromosome and calculated the correlation of expression of each possible gene pair using Pearson's correlation coefficient. We observed groups of coexpressed genes (*cor *≥ 0.5) on several chromosomes. As an example, the correlation matrix of human chromosome 10 shows areas of coexpressed genes between genes 7–10 and 27–30 [see Additional file 3]. Correlation maps provide a gross overview of the coexpression of genes located nearby each other. Therefore, we further analyzed the coexpression of gene pairs with respect to their distance on two different scales: the base pair distance and the number of genes located between pairs. For neither of these scales do we observe significant coexpression of co-localized gene pairs.

## Discussion

Our data suggest that a large proportion of the cardiac transcriptome in human is linearly arranged in small groups of adjacent genes such that the genes within each group tend to be regulated by the same transcription factors and appear to share granular biological processes. Even though the numbers of shared transcription factors and shared GO terms are small, they are considerable larger than what could be expected by chance. These findings provide powerful evidence that gene clustering plays a potentially important role in concerted gene regulation through the location of regulatory elements with respect of the regulated genes.

We focused our analysis on the information whether or not a gene and herewith a particular genomic region is transcribed at all in the human heart and did not consider rates of transcription. The proportion of genes arranged in clusters exceeded the number reported previously, which could be explained by different definitions of coexpression, as other reports defined coexpression mainly based on the correlation coefficient of continuous valued expression levels or considered only highly expressed genes [13]. Creating an expression neighborhood through the localization of regulatory elements would be an efficient means to increase regional gene activity, which has been shown to be influenced by the local concentration of regulatory proteins [24-26]. In addition, such regulatory elements may influence the expression status within a chromosomal region e.g. by spreading of histone modifications. Further support for concerted regulation of clustered genes is provided by our observation of functional relatedness between clustered genes. Apart from the identification of housekeeping genes arranged in clusters such relatedness has not been reported in human [14]. However without considering gene localization, it has been shown recently that coexpressed genes are often functionally related [21]. Further studies will be required to show if there is an evolutionary constraint upon the disruption of co-localized genes. So far, sequencing projects are still in process and a comparison between close relatives such as human and for example mouse would not be sufficient due to their high synteny. From our analysis, we propose that duplication events alone are not sufficient as an explanation.

In the past some reports suggested that tissue-specific genes, e.g. genes specifically expressed in human skeletal muscle and adipose tissue, are preferentially located on certain chromosomes [27,28]. For the cardiovascular transcriptome in human, a disparate chromosomal distribution has been reported with enrichment on chromosomes 17, 19 and 22 [29,30]. With the present knowledge of genome annotation that reveals the inhomogeneous chromosomal distribution of the human genome, we cannot confirm such observations for the cardiac transcriptome.

Finally, we analyzed correlation of expression levels of genes located in clusters, as it has been suggested for the human transcriptome and other organism. By using the base pair distance as well as the gene distance between gene pairs, we failed to observe any significant correlation between co-localization and expression levels. Recently, it has been proposed that such correlation could be influenced by the probe localization on the array [31], which was corrected for in our primary array analysis. Further, we took into account that our dataset was based on different cardiac phenotypes caused by multiple factors, which could increase background noise and reduce the ability to recognize distinct coexpression of genes in our sample collection.

## Conclusion

In summary, we provide evidence that the linear arrangement of genes expressed in concert is due to coordinated regulation by common transcription factors. We suggest that determining the relationship between nuclear organization and gene arrangement will lead to a deeper understanding of how transcriptomes, dedicated to a particular cellular function or fate, are controlled. Here, meta-analysis of the large-scale transcriptome array data beginning to appear in public repositories, could build the basis for the discovery of the nodes between gene regulation and nuclear organization. Those analyses could provide insights into a regulatory constraint such that genes localized in clusters tend to be coregulated throughout several tissues. Furthermore, such a regulatory constraint may be a crucial factor in the development of human diseases caused by partial deletions or insertions of chromosomal units separating genes regulated in concert.

## Methods

### HXP dataset

The data composition and the classification of expressed genes was done using the Human Unigene II clone set containing 74.695 IMAGE clones [22]. The genomic localizations of the clones were determined via Ensembl and CrossMatch sequence comparison. In summary, 40.416 clones were assigned to 16.260 Ensembl genes, which resulted in 67% coverage of the Ensembl dataset version 11.31.1. In a previous array study, we identified the cardiac transcriptome by hybridisation experiments of 55 cardiac samples using arrays containing the above IMAGE clones [22]. The finally defined heart-expressed subset (HXP) of IMAGE-clones contained 3.172 Ensembl genes represented by 4.167 clones. This gene set refers to the 15% highest expressed genes in at least 4 heart samples whose natural log ratios had a standard deviation across the samples of at least 0.5, and were obtained out of the overall set of human genes (HU2) after normalization of expression levels for array position and averaging over duplicates. The analyzed samples belonged to four categories, (1) the normal right/left atrial and ventricular samples, (2) right atrial and ventricular samples obtained from patients with Tetralogy of Fallot, (3) right atrial samples of patients with ventricular septal defect, and (4) right atrial samples of patients with atrial septal defect. For the further analysis, we excluded the Y chromosome since the dataset was composed of samples from males and females. Functional categories of these genes were assigned using the Gene Ontology classification.

### GFN Symatlas

To analyze the distribution of tissue expression of the HXP dataset, we used the microarray gene expression information for 79 human tissues in the GNF Symatlas dataset [15]. This dataset contained almost 34,000 probe sets with 'present', 'marginal', or 'absent' calls for each probe set in each tissue. We considered a probe set expressed if it had a 'present' or 'marginal' call. If probe sets with different expression calls had the same chromosomal location, we considered the 'present' or 'marginal' call in case where one of the probe sets had an 'absent' call. Probe Sets with ambiguous location information were excluded, such that the resulting dataset consisted of 10,715 Ensembl genes with distinct chromosomal locations. Based on the Ensembl gene IDs we could map 1,600 HXP genes including 183 clustered genes to expression information represented by the GNF Symatlas.

### Physical annotation of gene distances

To measure the distance of a pair of genes, we used the number of base pairs between them as well as defining the distance of a pair of genes with respect to the amount of Ensembl genes located between them (Fig. 1). A pair of genes was defined as adjacent if there are no other genes in the Ensembl dataset that lie between the two different gene loci. By using these different scales, we were able to distinguish between gene pairs located very close to each other in a chromosomal region with high gene density, as well as describing the relationship of a gene pair independent of its physical localization on the chromosome. Groups or pairs of directly neighboring genes that we analyzed are referred to as gene clusters. The definition of 'neighborhood' refers to the genes included in the Ensembl dataset.

### Chromosomal distribution of heart-expressed genes

The number of genes on each chromosome was calculated for the HXP and HU2 dataset. The two distributions were compared using the Chi-square test. The numbers of detected gene clusters were compared to a randomly built dataset based on the genes in the HU2 dataset (100,000 permutations). Furthermore, we used the hypergeometrical distribution to assess whether genes located close to each other share similar expression levels (*cor *≥ 0.65) more often than genes located further apart.

### Identification of regulatory transcription factors

The identification of putative regulatory transcription factors binding sites was done using the TRANSFAC database. To reduce the rate of false positives among putative binding sites, we first filtered our data by searching for conserved, non-coding upstream regions of orthologous genes in human and mouse as annotated in the *Comparative Regulatory Genomics database *CORG [23]. CORG is based on the assumption that high levels of sequence conservation in non-coding upstream regions of orthologous genes are likely to reflect common regulatory elements.

### Identification of similar gene expression levels

To determine similarity in gene expression for pairs of genes, we calculated the Pearson correlation coefficient as well as the Euclidian distance of their expression values across all 55 previously analyzed tissues [22]. To assess the potential relationship of gene localization and similar expression, we used distance-correlation plots and correlation matrices. Correlation matrices give a rough overview of similar expression levels of genes located on the same chromosome by displaying color-coded correlation coefficients (see for example web supplement Fig. 1S).

## Authors' contributions

JHV acquired the data and performed the main analysis of data. AvH has made substantive intellectual contribution to concept and interpretation of data. AP participated in analysis of data and drafting of article. SS conceived the project, managed and participated in analysis and interpretation of data.

## Supplementary Material

Additional File 1 **Transcription factors shared by gene clusters**. Represented are clustered heart-expressed genes with their HUGO gene names, Ensembl gene IDs, strand orientations, transcriptional start site distances and transcription factors for which binding sites could be predicted. The shared transcription factors are indicated in italic. Click here for file

Additional File 2 **Gene Ontology terms shared by gene clusters**. Shown are clustered heart-expressed genes annotated with Ensembl gene IDs, HUGO gene names and shared Gene Ontology (GO) terms of layer 4 and 5. The GO term IDs and descriptions are given. Click here for file

Additional File 3 **Correlation matrix of human chromosome 10**. The matrix of Pearson correlation coefficients between the expression profiles of heart-expressed genes is shown color-coded, with genes being arranged according to their order on the chromosome. Gene pairs with similar expression levels are depicted in blue, anti-correlated pairs are shown in red. Click here for file
