# Supplementary material for: Chromosomal clustering of a human transcriptome reveals regulatory background
Source: BMC Bioinformatics. 2005 Sep 19;6:230. doi: 10.1186/1471-2105-6-230 (PMC1261156; doi:10.1186/1471-2105-6-230)
Supplement: Additional File 1 — Transcription factors shared by gene clusters. Represented are clustered heart-expressed genes with their HUGO gene names, Ensembl gene IDs, strand orientations, transcriptional start site distances and transcription factors for which binding sites could be predicted. The shared transcription factors are indicated in italic. [file 1471-2105-6-230-S1.pdf]

Table 1S – Transcription factors shared by gene clusters

Cluster Nr	HUGO Name	Ensembl-ID	Strand	TSS Distance	Transcription Factor
1		ENSG00000127481	-		CDPCR3HD,TBP
1		ENSG00000127463	-	41304	AHRARNT,AML1,AP1,ARNT,COREBF,COUP,CP2,E12,ELF1,ETF,FOXO3,GKLF,LFA1,MYB,MYOD,RORA1,SMAD4,SRY,STAT5A,STAT5B,STAT6,TEF1
2		ENSG00000127463	-		AHRARNT,AML1,AP1,ARNT,COREBF,COUP,CP2,E12,ELF1,ETF,FOXO3,GKLF,LFA1,MYB,MYOD,RORA1,SMAD4,SRY,STAT5A,STAT5B,STAT6,TEF1
2	C1orf33	ENSG00000053372	+	253	HLF,USF
3		ENSG00000142687	-		*MZF1,YY1,* AP1,AP2ALPHA,AREB6,CEBP,CP2,EN1,FOXO4,GATA3,HAND1E47,LYF1,MEIS1,MSX1,NKX25,OCT1,PAX4,RREB1,SF1,TCF4,TGIF,TST1,VDR
3		ENSG00000020129	+	416	*MZF1,YY1,* AP2,AP2GAMMA,AP2REP,ATF,CACBP,GATA1,NFY,OSF2,PU1,SRY,STAT3
4	EIF2C4	ENSG00000134698	+		AP2ALPHA,FREAC2,LFA1,PAX4,PITX2,STAT3
4	EIF2C1	ENSG00000092847	+	61586	AML1,AREB6,CREBP1,ELF1,ETF,EVI1,FOXD3,FOXM1,FOXO1,HFH3,LEF1,LMO2COM,MYOGENIN,NFKAPPAB,NKX25,P53,PAX2,PEA3,PU1,SRY,STAT5A,STAT5B,STAT6
5	EIF2C1	ENSG00000092847	+		*AREB6,ETF,PU1,* AML1,CREBP1,ELF1,EVI1,FOXD3,FOXM1,FOXO1,HFH3,LEF1,LMO2COM,MYOGENIN,NFKAPPAB,NKX25,P53,PAX2,PEA3,SRY,STAT5A,STAT5B,STAT6
5	EIF2C3	ENSG00000126070	+	60910	*AREB6,ETF,PU1* , CETS1P54
6		ENSG00000116871	+		AP2,AP2GAMMA,AP4,AR,ATF1,BACH1,CDP,CDX2,CRX,E47,FOXM1,GATA4,HAND1E47,HEB,HNF6,HSF1,HSF2,IK3,ISRE,MAX,MEIS1,MMEF2,MYCMAX,NFE2,NFKAPPAB50,NKX62,NMYC,OCT1,OSF2,P300,PEA3,PITX2,PU1,RREB1,SMAD4,SOX5,SP1,SRY,STAT4,TCF11,TGIF,USF2,YY1
6	THRAP3	ENSG00000054118	+	68476	AREB6,P53,STAT1
7		ENSG00000085998	-		AML1,AP2GAMMA,AP2REP,ATF4,CACBP,HLF,IK1,LBP1,LMO2COM,NFKAPPAB65,NFY,SP1,TCF11,TCF1P,TFIIA,ZIC2,ZIC3
7		ENSG00000171357	+	16971	AP2ALPHA,CEBPB,FOXD3,GATA1,LYF1,STAT4
8		ENSG00000134748	+		AP2REP,AP3,AR,CEBPB,FOXJ2,LBP1,PAX4,POU1F1,SF1,STAT5B,TEF1,ZIC2
8		ENSG00000134744	-	148536	DBP,HMGIY,MEIS1,NFAT,NKX62,SRY
9		ENSG00000158966	+		*CEBPDELTA,IRF1,STAT5A,STAT6,TCF11,TCF4,* AML1,AP1,AP2ALPHA,AREB6,CDC5,CEBP,CEBPB,CETS1P54,HNF4ALPHA,HNF6,LBP1,PAX4,S8,SMAD3,VDR,YY1
9		ENSG00000162437	+	162936	*CEBPDELTA,IRF1,STAT5A,STAT6,TCF11,TCF4,* AP4,CACBP,CEBPGAMMA,FOXJ2,FOXM1,FREAC3,HFH3,HNF3ALPHA,HSF2,LHX3,MAZ,MZF1,NKX3A,NKX61,OCT1,P53,PAX2,PBX1,RREB1,SF1,SRY,TATA,TEF,TEF1
10	BCL10	ENSG00000142867	-		*AP2REP,FOXM1,GATA1,HOXA4,OCT1,PAX4,SOX5,STAT6,* AP4,FOXD3,GATA4,IRF1,LFA1,MEIS1,NFY,PEA3,STAT5B,TCF1P
10	DDAH1	ENSG00000153904	-	187145	*AP2REP,FOXM1,GATA1,HOXA4,OCT1,PAX4,SOX5,STAT6,* AREB6,CDX2,CETS1P54,E12,E2F1,FOXO4,HFH3,HMGIY,MAZ,MYOD,NKX25,PAX8,PBX1,POU1F1,POU3F2,RP58,S8,STAT1,STAT4,TITF1,USF2,ZIC2
11		ENSG00000162641	-		*AP2REP,CEBP,* CDC5,P53,PITX2,PU1
11	GPSM1	ENSG00000121957	+		*AP2REP,CEBP,* AP3,ARP1,CEBPGAMMA,FAC1,HLF,HMGIY,LYF1,SMAD3,STAT5A
12		ENSG00000023902	+		CREL,CRX,DBP,HFH3,HOXA3,LFA1,MYB
12	ANP32E	ENSG00000143401	-	86336	AP2ALPHA,ATF1,BRN2,CEBPDELTA,ERR1,GKLF,HSF2,IPF1,MZF1,NFAT,NFE2,NFY,PAX2,SOX5,TEF1
13	BNIPL	ENSG00000163141	+		AP4,DBP,HAND1E47,LYF1,MAZ,RP58,SRY,TCF1P,YY1
13		ENSG00000175498	unknown		AP1,CEBPB,HEB
14		ENSG00000175498	unknown		AP1,CEBPB,HEB
14		ENSG00000143415	-		AP2REP,AP4,GKLF,MAZ,NKX25,PAX4,PU1,SRY,STAT3,TEF,TFIII
15	TUFT1	ENSG00000143367	+		*HNF4ALPHA,TATA,* CACCCBF,ETF,MYOGENIN,OSF2,SF1
15	SNX27	ENSG00000143376	+	107458	*HNF4ALPHA,TATA,* COUP,DBP,FOXM1,IK2,LYF1,OCT1,STAT6,TBP
16	PEA15	ENSG00000162734	+		*AML1,AP1,CDPCR3HD,CEBPB,CRX,EVI1,HOXA4,P53,RORA2,TCF1P,TST1,USF2,ZIC3* , AP2ALPHA,AREB6,BACH1,BACH2,CDC5,COREBF,E2F1,GATA1,GKLF,HNF4ALPHA,HSF1,IK2,MYOD,NCX,OCT1,PAX4,PU1,SOX5,STAT,TFIIA
16	WDR42A	ENSG00000132716	-	57112	*AML1,AP1,CDPCR3HD,CEBPB,CRX,EVI1,HOXA4,P53,RORA2,TCF1P,TST1,USF2,ZIC3* , CEBP,GATA3,GATA6,HLF,HMEF2,LFA1,NFAT,NFY,NKX25,NKX62,PBX1,STAT1,STAT5A,TBP,TEF
17	WDR42A	ENSG00000132716	-		AML1,AP1,CDPCR3HD,CEBP,CEBPB,CRX,EVI1,GATA3,GATA6,HLF,HMEF2,HOXA4,LFA1,NFAT,NFY,NKX25,NKX62,P53,PBX1,RORA2,STAT1,STAT5A,TBP,TCF1P,TEF,TST1,USF2,ZIC3
17	PEX19	ENSG00000162735	-	22686	SRY
18		ENSG00000117477	-		*PEA3,* SRY,TAL1ALPHAE47,TCF4
18	SLC19A2	ENSG00000117479	-	58529	*PEA3,* AML1,AP1,AP4,CETS1P54,COREBF,CREBP1,CREL,DBP,ETF,FOXO4,GATA1,GATA3,GATA4,MMEF2,MSX1,MYB,MYOD,NKX22,P53,PITX2,RORA1,SMAD3,SP1,STAT5A,STAT5B,TBP,TFIIA
19		ENSG00000135835	+		*AP2REP,TEF1,* AP3,CACBP,HMGIY,HNF1,ICSBP,NFAT,PU1,STAT6,ZF5
19	STX6	ENSG00000135823	-	92273	*AP2REP,TEF1,* AREB6,BACH2,CACCCBF,CDX2,CEBPB,EN1,HNF3ALPHA,LEF1,MMEF2,MYOD,NKX25,TFIII,YY1
20		ENSG00000120334	-		DBP,PBX1,TCF4
20		ENSG00000117593	+	150	CEBPB,E2F1,E2F1DP1,ETF,GATA1,HEB,IRF1,LYF1,NFAT,NKX22,PU1,YY1,ZF5
21	PROX1	ENSG00000117707	+		E2F1DP1,HLF,NKX25,OCT1,PEA3,SRY,TBP,USF
21	SMYD2	ENSG00000143499	+	284696	AP3,AREB6,CEBPGAMMA,EN1,ERR1,FOXM1,MYOGENIN,PAX2,PU1,SOX5,STAT1,STAT4,STAT5A,YY1
22	ACTN3	ENSG00000077522	+		*AP4,LYF1,* AML1,AREB6,CDP,CEBP,CEBPGAMMA,CREBP1CJUN,ELF1,FAC1,FOXD3,FOXJ2,IK3,MYB,NKX25,NKX61,OCT1,POU1F1,SMAD3,STAT5B,TBP,TEF,ZIC3
22	MTR	ENSG00000116984	+	108915	*AP4,LYF1,* E2F1,LBP1,MEIS1,ZF5
23	HNRPU	ENSG00000153187	-		*LYF1,MAZ,STAT5A,STAT5B,* AHRARNT,AP2GAMMA,E2F1DP1RB,FOXD3,HNF3ALPHA,NFE2,NKX3A,P53,PAX4,TCF1P
23		ENSG00000153188	+	105460	*LYF1,MAZ,STAT5A,STAT5B,* AML1,AP1,AP2ALPHA,AP3,AREB6,ARP1,BRN2,CACBP,CEBPDELTA,CREBP1CJUN,ELF1,ETF,EVI1,FOXO4,GATA4,GCM,HMGIY,HNF6,IRF1,LHX3,MEIS1,MRF2,MSX1,MYB,NFAT,NKX62,OCT1,PBX1,POU1F1,RP58,STAT1,TEF,TEF1,TFIIA,USF,ZID
24	HADHA	ENSG00000084754	-		*PITX2,TCF4,* AP1,HOXA4,MYOGENIN,SOX5
24	HADHB	ENSG00000138029	+	295	*PITX2,TCF4,* CEBPDELTA,FREAC4,IRF1,MZF1,SRY,STAT5A,STAT6,TITF1
25	SPG4	ENSG00000021574	+		*SRY,* FOXD3,FOXM1,OCT1,OSF2,PAX2,RORA1,TCF1P,USF2,ZF5,ZIC3
25	SLC30A6	ENSG00000152683	+	102247	*SRY,* AP2REP,DBP,HMGIY,HOXA3,NKX3A,POU6F1
26	XPO1	ENSG00000082898	-		*LYF1,* AR,CDP,CEBPB,E12,FAC1,HOXA4,HSF2,IRF1,MYB,MYOD,PAX8,PITX2,SMAD3,SRY,TITF1
26		ENSG00000170264	-	315848	*LYF1,* AML1,AP1,AP2ALPHA,AP2REP,LFA1,MAZ,NKX25,NKX61,PAX2,RORA1,RORA2,STAT5B,STAT6,TATA,TEF,TFIII
27	UGP2	ENSG00000169764	+		AP1FJ,AP2GAMMA,AP2REP,AP4,AREB6,CACBP,CEBPA,DBP,E2F,FOXD3,GATA3,IK2,LBP1,LYF1,MMEF2,MYB,MYCMAX,MZF1,NFAT,NFY,S8,STAT,TEF1
27	VPS54	ENSG00000143952	-	142935	HMGIY,HOXA4,NKX25,TCF4,YY1
28	MAD	ENSG00000059728	+		*GKLF,P53,PU1,TBP,* AP2ALPHA,ATF3,HOXA4,LFA1,MZF1,NFY,PAX2,PEA3,TCF11
28		ENSG00000179818	-	47080	*GKLF,P53,PU1,TBP,* AML1,AP1,AP1FJ,ATF4,CETS1P54,DBP,ELF1,EN1,ETF,FOXO4,HFH3,HMGIY,HNF1,LEF1,OCT1,OSF2,RREB1,SF1,SP1,SRY,STAT5A,STAT5B,TCF4,VDR
29		ENSG00000135956	-		*AP2REP,ATF4,DBP,ELK1,GATA3,HEB,IK3,TCF1P,* CEBPA,CREL,E2F,ERR1,MYOGENIN,P300,STAT6,ZIC3
29		ENSG00000144021	+	197	*AP2REP,ATF4,DBP,ELK1,GATA3,HEB,IK3,TCF1P,* AP2ALPHA,AP4,AREB6,CEBPB,COUP,EN1,GATA4,LFA1,MAZ,MZF1,PAX4,PEA3,SPZ1,SRY,STAT5A,STAT5B,TAXCREB,TCF4,TITF1,USF2,ZIC1
30		ENSG00000144021	+		*AP2REP,AREB6,SRY,* AP2ALPHA,AP4,ATF4,CEBPB,COUP,DBP,ELK1,EN1,GATA3,GATA4,HEB,IK3,LFA1,MAZ,MZF1,PAX4,PEA3,SPZ1,STAT5A,STAT5B,TAXCREB,TCF1P,TCF4,TITF1,USF2,ZIC1
30		ENSG00000144028	-	39355	*AP2REP,AREB6,SRY,* EVI1,MSX1,PU1,ZF5
31	PTPN4	ENSG00000088179	+		AP1,AP3,CEBP,DBP,EVI1,IRF1,NKX22,NKX25,STAT6,TEF1
31	EPB41L5	ENSG00000115109	+	253446	CACBP,CDX2,E47,LMO2COM,LYF1,MZF1,TCF4
32	EPB41L5	ENSG00000115109	+		*CACBP,LMO2COM,TCF4,* CDX2,E47,LYF1,MZF1
32	FAM11B	ENSG00000179724	-	210331	*CACBP,LMO2COM,TCF4,* AP1FJ,CEBPGAMMA,DBP,IK2,LFA1,MYOD,NFE2,OSF2,PAX4,PEA3,PU1,SRY,STAT6,TFIII,TITF1,USF
33	FAM11B	ENSG00000179724	-		*AP1FJ,STAT6,* CACBP,CEBPGAMMA,DBP,IK2,LFA1,LMO2COM,MYOD,NFE2,OSF2,PAX4,PEA3,PU1,SRY,TCF4,TFIII,TITF1,USF
33	RALB	ENSG00000144118	+	29445	*AP1FJ,STAT6,* AP4,CETS1P54,EVI1,GATA1,HMGIY,MZF1,NFAT,P300,STAT5A,TBP,USF2,ZIC2
34	PREI3	ENSG00000115540	+		*STAT5A,* GATA3,PITX2,PU1,STAT1,STAT3,TFIII
34	C2orf11	ENSG00000162944	-	159817	*STAT5A,* ATF6,ERR1,GATA1,LBP1,NCX,NKX25,OCT1,SMAD4
35	RAPH1	ENSG00000173166	-		*AP3,EVI1,FOXM1,MSX1,TBP,TCF1P,* AFP1,AR,DBP,ERR1,FOXD3,HAND1E47,HSF1,IRF1,MMEF2,MYOD,NKX3A,NKX62,PITX2,POU3F2,PU1,SRY,STAT4,STAT5B,TCF4,ZIC2
35	CD28	ENSG00000178562	+	171150	*AP3,EVI1,FOXM1,MSX1,TBP,TCF1P,* AML1,AP2GAMMA,GATA1,GATA4,GKLF,HLF,LFA1,MEIS1,NFY,OCT1,PAX2,POU6F1,SMAD3,SREBP1,STAT6,TATA,TFIIA,ZID
36	CD28	ENSG00000178562	+		*AP3,EVI1,GATA1,LFA1,MEIS1,OCT1,STAT6,TFIIA,* AML1,AP2GAMMA,FOXM1,GATA4,GKLF,HLF,MSX1,NFY,PAX2,POU6F1,SMAD3,SREBP1,TATA,TBP,TCF1P,ZID
36	CTLA4	ENSG00000163599	+	161286	*AP3,EVI1,GATA1,LFA1,MEIS1,OCT1,STAT6,TFIIA,* AFP1,AP2ALPHA,AP4,CEBPB,COREBF,CRX,E2F1,ELF1,ERR1,FAC1,HNF3ALPHA,HOXA4,HSF2,IPF1,LEF1,LMO2COM,MEIS1BHOXA9,MMEF2,MYOGENIN,MZF1,NKX25,NKX61,P53,PBX1,PEA3,RORA2,SMAD4,SP1,SRY,STAT5A,TEF1,TGIF,USF
37	CREB1	ENSG00000118260	+		AREB6,ATF1,CEBPGAMMA,CREL,FOXM1,GATA4,HNF4ALPHA,HNF6,IRF1,LBP1,MEIS1,POU1F1,RREB1,STAT,TBP,TEF1,TST1,ZIC2
37		ENSG00000144401	-	95369	AP2REP,CRX
38	FBLN2	ENSG00000163520	+		*AP2REP,* AP4,ETF,GATA1,IK2,MYCMAX,MZF1,NFAT,OSF2,POU1F1,PU1,SF1,STAT3,STAT4,TCF4,ZIC2
38	WNT7A	ENSG00000154764	-	309809	*AP2REP,* CREB,LMO2COM,MAZ,NKX25,OCT1,TCF11,ZF5
39	GALNTL2	ENSG00000131386	+		*EN1,MZF1,* CACBP,CEBPB,CETS1P54,COREBF,CREB,CRX,GATA2,HEB,HMGIY,LBP1,MEIS1,MYOGENIN,NFE2,NFKB,SMAD4,SOX5,TAL1BETAE47,TBP,TITF1,ZIC3
39	ZCSL2	ENSG00000154813	-	90293	*EN1,MZF1,* AP2ALPHA,AP2REP,ERR1,FAC1,GATA4,STAT5A,TCF4
40	TGM4	ENSG00000163810	+		*STAT5A,* SRY,TEF1
40	ZDHHC3	ENSG00000163812	-	101511	*STAT5A,* AML1,AP2REP,AREB6,CETS1P54,CHOP,DBP,E2F1,FOXO1,FOXO4,GATA1,GATA4,HFH4,HMGIY,HOXA3,HOXA4,IK2,IRF1,LFA1,LHX3,NKX25,PAX4,PAX8,POU1F1,SMAD3,SMAD4,STAT3,STAT6,TST1,USF,USF2
41	KIF9	ENSG00000088727	-		AP1,CREB,ELF1,ETF,GATA3,GCM,HMGIY,LBP1,LFA1,LYF1,NFKB,PU1,TFIII
41	KLHL18	ENSG00000114648	+	273	STAT5A
42	SEMA3B	ENSG00000012171	+		*IPF1,* FOXO4,LBP1,LFA1,MEIS1
42	GNAI2	ENSG00000114353	+	18191	*IPF1,* AML1,AP1,AP2GAMMA,AREB6,CDC5,CDP,CEBP,CEBPA,CHOP,COREBF,COUP,CRX,DBP,EFC,ELF1,ERR1,EVI1,HSF1,LHX3,LMO2COM,PITX2,SMAD3,SMAD4,STAT,STAT5A,TCF1P,ZIC3
43	PXK	ENSG00000168297	+		*HMGIY,* AP2GAMMA,AP3,COREBF,DBP,EN1,FAC1,GATA1,HOXA3,ICSBP,LEF1,MAZ,MYCMAX,NFY,NKX3A,OCT1,PEA3,STAT6,TCF4,TGIF,TST1,USF2
43	PDHB	ENSG00000168291	-	100937	*HMGIY,* AREB6,CACBP,MYB,SRY
44	PVRL3	ENSG00000177707	+		*CEBP,E2F1,LBP1,MZF1,* AP2REP,ETF,HOXA4,LMO2COM,MAZ,NFAT,PU1,SP1,STAT5A
44	CD96	ENSG00000153283	+	470448	*CEBP,E2F1,LBP1,MZF1,* AP1,AREB6,ATF,CDX2,CEBPGAMMA,CREL,E12,EN1,GATA2,HFH3,LHX3,LYF1,MYB,OCT1,PAX2,POU1F1,TBP,TCF1P,TCF4,TEF1,TFIII,USF2,VDR
45	PHLDB2	ENSG00000144824	+		AP2GAMMA,AP3,AP4,AREB6,CDP,CEBP,CEBPB,CEBPGAMMA,CREBP1,DBP,E12,FOXM1,GATA1,GATA6,HNF1,ICSBP,IK2,LEF1,LYF1,MAZ,MEIS1,MYCMAX,MYOD,MYOGENIN,NFAT,OCT1,PAX2,PAX4,PEA3,SOX5,SP3,SRY,STAT,STAT6,TBP,TEF1,TGIF,USF2,YY1
45		ENSG00000144827	+	119277	AP2REP,GATA4,HSF2,NFKAPPAB,SF1,STAT4
46		ENSG00000138496	-		*DBP,STAT4,STAT6,* AP2REP,ELF1,EN1,LFA1,LYF1,NFAT,STAT5A
46		ENSG00000163840	+	171	*DBP,STAT4,STAT6,* AP2ALPHA,CRX,GATA3,HMGIY,IRF1,PEA3,SRY,STAT5B,TCF4
47	PARP14	ENSG00000173193	+		AP1,AP2ALPHA,AREB6,ETF,FOXJ2,GATA1,GATA3,HEB,HMGIY,HOXA4,LHX3,MEF2,MEIS1,MYOD,NFY,OSF2,PAX4,PEA3,POU6F1,SRF,STAT5A,TEF1,TITF1,USF2,ZID
47	HSPBAP1	ENSG00000169087	-	94227	CACBP,E2F1,HSF2,SREBP1,SRY
48	PPP2R3A	ENSG00000073711	+		*SF1,SRY,* AP4,AREB6,CACBP,CDPCR3HD,CEBPGAMMA,FOXM1,GATA1,HMGIY,IRF1,LFA1,MEIS1,MSX1,MYCMAX,NFE2,SPZ1,STAT,STAT6,TITF1
48		ENSG00000174579	-	230142	*SF1,SRY,* AP1,AP2ALPHA,AR,CDX2,CEBP,E2F,E2F1,FAC1,FOXO4,HFH3,HFH8,HOXA3,IK2,LBP1,NKX22,NKX3A,P53,PAX8,PEA3,POU1F1,STAT5A,STAT5B,TCF4,ZIC1,ZID
49	DBR1	ENSG00000138231	-		*HOXA4,NFAT,NKX25,NKX61,* CEBPGAMMA,EN1,GKLF,HFH8,HNF1,IK2,MMEF2,MRF2,PAX2,PAX4,POU1F1,STAT1,STAT5A,ZIC1

49		ENSG00000114098	+	12644	*HOXA4,NFAT,NKX25,NKX61,* AREB6,CACBP,DBP,EVI1,FOXO4,GATA3,GATA6,HFH4,HLF,HOXA3,LFA1,LYF1,MYOGENIN,OCT1,OSF2,P53,PEA3,PITX2,STAT5B,STAT6,TCF4,TFIII,TST1
50	ARMC8	ENSG00000114790	+		*STAT5A,* AP1,AP2REP,AP3,AR,AREB6,ATF3,CEBPDELTA,FOXD3,GATA4,HFH3,HLF,HSF1,MMEF2,MYB,OCT1,PAX4,SP1,STAT6,TCF4,ZIC3
50	DHX36	ENSG00000174953	-	203081	*STAT5A,*
51		ENSG00000068885	-		AP1FJ,AP3,AP4,DBP,E47,EVI1,FOXJ2,HFH3,HMGIY,HOXA4,MSX1,NKX25,NKX62,PU1,STAT,STAT1,STAT5A,TAL1BETAITF2,TCF4,YY1
51	SMC4L1	ENSG00000113810	+	441	MYB
52	DVL3	ENSG00000161202	+		*CETS1P54,* AP3,CP2,MZF1,STAT3
52	AP2M1	ENSG00000161203	+	19467	*CETS1P54,* AP2REP,AREB6,ATF1,ATF4,FOXM1,FOXO1,HNF4ALPHA,LFA1,LYF1,MAZ,MYOGENIN,NKX62,P53,RORA2,SMAD3,TFIIA,ZIC2
53	C4orf9	ENSG00000087269	-		AR,HEB,RSRFC4
53	GRK4	ENSG00000125388	+	246	E2F1,FOXO1,NFAT,STAT5B,ZF5
54	GRK4	ENSG00000125388	+		E2F1,FOXO1,NFAT,STAT5B,ZF5
54	HD	ENSG00000125387	+	110888	SF1,STAT6
55	ZCCHC4	ENSG00000168228	+		E2F1,HOXA4,IRF1,TCF4,USF
55	ANAPC4	ENSG00000053900	+	64632	AP1,CEBPGAMMA,CREBP1CJUN,DBP,FOXM1,HNF3ALPHA,HNF6,IPF1,MYOD,NKX25,OCT1,PU1,SF1,SRY,STAT5A,STAT6,TEF1,USF2
56		ENSG00000180609	unknown		*CACBP,* CDX2,GATA3
56	EPHA5	ENSG00000145242	-		*CACBP,* AP1,AP2,AP2GAMMA,AP3,AP4,AREB6,BACH2,CDPCR3HD,CEBPA,CEBPDELTA,CHX10,DBP,E2F1,ERR1,FOXD3,FOXM1,FOXO4,FREAC3,GATA6,GKLF,HAND1E47,HEB,HMGIY,HNF1,IRF1,LFA1,MAZ,MYB,MZF1,NKX62,OCT1,PAX8,RP58,SF1,SP1,SP3,TST1,USF,USF2
57	PRKAA1	ENSG00000132356	-		AP2GAMMA,ELF1,IRF1,MAZ,PAX2,SF1,SP1,SRY,STAT6,TITF1
57	RPL37	ENSG00000145592	-	37029	CEBPGAMMA,MYOD,STAT5B
58	RPL37	ENSG00000145592	-		CEBPGAMMA,MYOD,STAT5B
58	CARD6	ENSG00000132357	+	5976	LFA1,P53,USF2
59	CARD6	ENSG00000132357	+		LFA1,P53,USF2
59	C7	ENSG00000112936	+	68227	CEBP,DBP,GATA6,GKLF,HEB,LYF1,MYOD,NFAT,SF1,SP1,TEF1
60	MSH3	ENSG00000113318	+		*SF1,STAT6,* AML1,AP3,TEF1,TITF1
60	RASGRF2	ENSG00000113319	+	305769	*SF1,STAT6,* AP2GAMMA,AP4,AREB6,CDC5,CHOP,ELF1,EN1,HMGIY,IRF1,LYF1,MAZ,MYOGENIN,NFAT,NKX25,NKX3A,NMYC,OCT1,PAX2,PEA3,STAT,TATA,TBP,TCF1P,TGIF,USF
61	RASGRF2	ENSG00000113319	+		*AREB6,EN1,PAX2,PEA3,* AP2GAMMA,AP4,CDC5,CHOP,ELF1,HMGIY,IRF1,LYF1,MAZ,MYOGENIN,NFAT,NKX25,NKX3A,NMYC,OCT1,SF1,STAT,STAT6,TATA,TBP,TCF1P,TGIF,USF
61	CKMT2	ENSG00000131730	+	272632	*AREB6,EN1,PAX2,PEA3,* CDPCR1,CDPCR3,FOXJ2,FOXO1,HNF3ALPHA,MTF1,P53,PAX4,PBX1,STAT4,STAT5A,TCF11
62	CAST	ENSG00000153113	+		*HMGIY,LYF1,* CEBPB,CEBPGAMMA,DBP,EVI1,HEB,HNF3ALPHA,LBP1,NKX25,OCT1,TEF,USF2
62		ENSG00000164307	-	132401	*HMGIY,LYF1,* AREB6,HNF4ALPHA,HOXA4,ICSBP,LFA1,OSF2,P53,PU1,STAT6,TCF1P,TEL2,ZIC2
63		ENSG00000164403	-		*SMAD4,* AP1FJ,AP2REP,AP4,CACBP,CDPCR1,CP2,E2F,FAC1,HEB,HOXA3,ICSBP,LFA1,NKX25,SOX5,SRY,STAT1,TFIII,ZIC1,ZIC3
63	GDF9	ENSG00000164404	-	33636	*SMAD4,* AP1,GATA2,IRF1,LYF1,MYOGENIN,SF1,STAT4,ZIC2
64	GDF9	ENSG00000164404	-		*IRF1,* AP1,GATA2,LYF1,MYOGENIN,SF1,SMAD4,STAT4,ZIC2
64		ENSG00000164405	+	2136	*IRF1,* AP2ALPHA,GATA4,HFH4,LEF1,OSF2,TCF1P
65	SARA2	ENSG00000152700	-		*AREB6,DBP,GKLF,SF1,SRY,TBP,* AP1,AP3,CACBP,CEBPA,COUP,CREB,FOXD3,FOXM1,HLF,HMGIY,HOXA4,LFA1,LMO2COM,SP3,TEF1,USF2
65	SEC24A	ENSG00000113615	+	15983	*AREB6,DBP,GKLF,SF1,SRY,TBP,* AP2GAMMA,ARNT,ATF6,CEBPB,CP2,ETF,GATA6,MYOD,NCX,NFKAPPAB65,PAX4,PBX1,RORA1,SMAD3,SOX5,TGIF,TITF1,USF,ZID
66	SEC24A	ENSG00000113615	+		AP2GAMMA,AREB6,ARNT,ATF6,CEBPB,CP2,DBP,ETF,GATA6,GKLF,MYOD,NCX,NFKAPPAB65,PAX4,PBX1,RORA1,SF1,SMAD3,SOX5,SRY,TBP,TGIF,TITF1,USF,ZID
66	CAMLG	ENSG00000164615	+	89694	AP3,LBP1
67	NDUFA2	ENSG00000131495	-		*AML1,AP2REP,ZIC1,* AP1,AP2GAMMA,AP3,CEBPB,E2F1,E47,E4F1,ERR1,FOXM1,GATA2,LMO2COM,MYOGENIN,NFY,OCT1,POU6F1,SMAD3,SRY,TBP,TFIII
67	IK	ENSG00000113141	+	140	*AML1,AP2REP,ZIC1,* ETF,MZF1,SPZ1,STAT5B
68	IK	ENSG00000113141	+		AML1,AP2REP,ETF,MZF1,SPZ1,STAT5B,ZIC1
68		ENSG00000120314	+	17054	AP1FJ,AREB6,LBP1,PITX2,POU1F1,TCF4,TFIII
69	DIAPH1	ENSG00000131504	-		AP2REP,LYF1,PAX2,TEF1
69	HDAC3	ENSG00000171720	-	17931	AML1,AP1,AP2,AP4,AREB6,ATF,ATF3,CP2,E47,GATA1,HSF2,MYCMAX,NFMUE1,PAX4,SP1,SREBP1,STAT3,STAT5A,STAT5B,TAL1ALPHAE47,TCF11,TCF4,TITF1,USF,VDR,ZIC1,ZIC3
70	SYNPO	ENSG00000171992	+		*AML1,FOXO4,GATA3,SP1,* AP2ALPHA,AP4,ATF3,ETF,GCM,HMGIY,MAZ,MYB,MYOD,MZF1,PAX2,S8,SF1,SMAD3,SMAD4,SPZ1,TATA,TST1
70	MYOZ3	ENSG00000164591	+	60257	*AML1,FOXO4,GATA3,SP1,* BACH2,CETS1P54,E12,E2F,FREAC2,HEB,LYF1,NMYC,PAX4,PAX8,PITX2,SRF,STAT5B,ZID
71	HMMR	ENSG00000072571	+		*TCF4,* AML1,AP1,AR,AREB6,BRN2,CEBP,CETS1P54,EN1,FAC1,FOXD3,FXR,MYOD,NFY,OCT1,PITX2,POU1F1,STAT6,TBP,TFIIA,ZIC1
71	MAT2B	ENSG00000038274	+	42602	*TCF4,* AP2REP,MRF2,MSX1,NKX25,PAX2,PAX8,SMAD3,SRY
72		ENSG00000124796	unknown		*DBP,GATA6,TBP,* AREB6,CACBP,CEBPGAMMA,CHX10,EN1,FAC1,FOXD3,GKLF,MRF2,MZF1,NKX25,NKX61,SMAD3,SRY,STAT4,STAT5A,TCF4
72	EEF1E1	ENSG00000124802	-		*DBP,GATA6,TBP,* AFP1,E12,FREAC7,GATA4,MSX1,NKX62,P53,PEA3,SP1
73	EEF1E1	ENSG00000124802	-		*TBP,* AFP1,DBP,E12,FREAC7,GATA4,GATA6,MSX1,NKX62,P53,PEA3,SP1
73	SLC35B3	ENSG00000124786	-	332844	*TBP,* EN1,HAND1E47,IK1,MYB,MZF1,NFE2,OCT1,POU6F1,STAT5B,TCF4,TEF1,YY1
74	KIF13A	ENSG00000137177	-		*STAT6,* AP2REP,AP4,CEBPGAMMA,CETS1P54,ETF,FOXM1,GATA6,IRF1,OCT1,SF1,TITF1,VDR,YY1,ZIC2
74	TPMT	ENSG00000137364	-	167592	*STAT6,* DBP,POU1F1
75	KIAA1117	ENSG00000083097	+		*AML1,LEF1,* AP1,AP3,BACH2,CREBP1,ERR1,EVI1,FOXD3,FOXJ2,FOXO1,GATA3,GATA4,GCM,HAND1E47,HMGIY,LMO2COM,MRF2,MYOD,NFAT,NKX3A,OCT1,P300,P53,POU1F1,PU1,RP58,SF1,SPZ1,STAT,STAT5A,STAT5B,STAT6,TBP,TCF11,TEF1,ZIC1
75	PGM3	ENSG00000013375	-	70388	*AML1,LEF1,* EN1,ETF
76	UBE2J1	ENSG00000111892	-		ATF6,ETF,HFH3,NFY,NKX25,SP1,TCF4
76	RRAGD	ENSG00000025039	-	59211	COREBF,DBP,ELF1,IRF1,PITX2,SRY,STAT5B,TFIII
77	RRAGD	ENSG00000025039	-		*SRY,* COREBF,DBP,ELF1,IRF1,PITX2,STAT5B,TFIII
77	ANKRD6	ENSG00000135299	+	154762	*SRY,* E12,LFA1,NKX25,TITF1
78	RTN4IP1	ENSG00000130347	-		*HNF6,HOXA4,LYF1,OCT1,SOX5,SRY,STAT5B,TEF1,* AREB6,FOXM1,GATA4,HLF,MEIS1,PEA3,SF1,STAT3,USF
78	QRSL1	ENSG00000130348	+	285	*HNF6,HOXA4,LYF1,OCT1,SOX5,SRY,STAT5B,TEF1,* CRX,ETF,HMGIY,PBX1,STAT4,TBP
79	PLN	ENSG00000178532	+		*AP3,NFAT,STAT5A,* AP4,EVI1,MZF1,NKX3A,PEA3,SRY,TBP,TCF4,TEF,TEF1,TGIF
79	C6orf61	ENSG00000178346	-	267364	*AP3,NFAT,STAT5A,* AP2REP,CEBPB,CEBPGAMMA,E2F1,FOXJ2,FOXM1,FOXO4,HOXA4,IRF1,LFA1,MYOD,MYOGENIN,NFE2,NKX25,NRF1,P53,STAT1,TITF1,USF2
80	SYNJ2	ENSG00000078269	+		FOXO1
80	SERAC1	ENSG00000122335	-	186387	AP2GAMMA,AP3,HSF1,IPF1,MYB,TBP
81	OGDH	ENSG00000105953	+		*DBP,LFA1,MAZ,SRY,TEF1,* AML1,AR,CEBP,CEBPGAMMA,CRX,FOXM1,HFH3,MSX1,PITX2,SMAD3,STAT5A,TATA,TEF,TITF1
81		ENSG00000122515	+	149597	*DBP,LFA1,MAZ,SRY,TEF1,* CP2,CREB,CREL,E2F1,ELF1,GATA1,GATA2,HIF1,LBP1,LEF1,MYCMAX,PAX4,PAX8,SF1,SP1,SPZ1,USF,ZID
82	FKBP6	ENSG00000077800	+		*E4BP4,TFIIA,* CP2,LEF1,SF1
82	BAZ1B	ENSG00000009954	-	194252	*E4BP4,TFIIA,* DBP,E12,HOXA4,LFA1,MYB,NCX,NFKB,NFY,PAX4,SP1,TCF4
83		ENSG00000001629	+		*DBP,PU1,TBP,TEF1,* AML1,AP2REP,CEBPB,FOXO1,GATA3,GATA4,HNF6,IRF1,LYF1,MYOGENIN,NFAT,NKX25,P300,STAT4,ZF5
83		ENSG00000157259	+	201243	*DBP,PU1,TBP,TEF1,* CEBPDELTA,FOXO4,HFH8,HIF1,HNF1,NKX3A,OCT1,TCF1P,USF2
84	PEX1	ENSG00000127980	-		*EVI1,* AML1,AP2REP,AREB6,CEBP,CEBPB,EN1,FOXO3,IPF1,MMEF2,NKX22,P300,PU1,SRY,TEF1,TST1
84		ENSG00000127993	+	300	*EVI1,* AP4,ATF3,BACH2,COREBF,GATA2,HEB,HFH8,HLF,HNF3ALPHA,HOXA3,IK1,LBP1,LMO2COM,LYF1,NKX25,OCT1,P53,PAX4,TATA,TBP,TCF11,TCF1P,TEF
85	BET1	ENSG00000105829	-		*FOXM1,HMGIY,OCT1,PITX2,* CDC5,MEIS1,NKX3A,P300,TST1
85	COL1A2	ENSG00000164692	+	390671	*FOXM1,HMGIY,OCT1,PITX2,* AML1,AP1,AP2GAMMA,AREB6,DBP,FREAC7,GATA1,HNF3ALPHA,HOXA4,MAZ,MRF2,NCX,NKX25,NKX61,POU6F1,PU1,S8,SF1,SPZ1,STAT3,TCF1P,TEF,TEF1
86	ARHGEF10	ENSG00000104728	+		AP2ALPHA,AP2REP,HLF,IRF1,LFA1,MEIS1,PAX2,PU1,SP1,SRY,STAT3
86		ENSG00000176575	unknown		DBP,EN1,GCM,RREB1,STAT5A
87	LYSAL1	ENSG00000167031	-		*LFA1,* AP1,AREB6,BACH2,CEBPDELTA,EN1,FOXD3,FOXM1,GATA3,HOXA3,HSF2,PAX2,PEA3,PITX2,POU1F1,TCF4,VDR,ZF5,ZIC3
87		ENSG00000147454	+	71304	*LFA1,* AP3,AP4,EVI1,MYB,MYOD,OCT1,PU1,SOX5,STAT5B,YY1
88		ENSG00000085788	+		*CDPCR3HD,DBP,LFA1,OCT1,YY1,* AP3,AREB6,CACBP,EN1,GKLF,HOXA3,HOXA4,ICSBP,LBP1,LYF1,MEIS1,MYB,NKX3A,PU1,SOX5
88		ENSG00000147535	-	37590	*CDPCR3HD,DBP,LFA1,OCT1,YY1,* AP2GAMMA,CETS1P54,CREL,CRX,HLF,MSX1,MZF1,NFY,OSF2,POU6F1,TCF11,TEF1
89	SGKL	ENSG00000104205	+		AREB6,DBP,FOXO1,HOXA3,NFAT,SOX5,SRY,STAT,STAT1,TFIII,TITF1
89		ENSG00000178460	+	83668	AFP1,CDPCR3HD,COREBF,ETF,GATA2,GATA3,HMEF2,IK1,MEIS1,NKX62,SF1,STAT6,TBP,TCF4,ZIC2
90		ENSG00000164764	-		*MAZ,ZF5,* AML1,AP1,CDX2,CP2,CRX,EN1,FOXD3,FOXO1,GATA3,HSF1,MRF2,OCT1,PAX4,PEA3,RORA1,SF1,STAT5A,STAT6,TCF4,TEF1,TITF1
90	RPL7	ENSG00000147604	-	169907	*MAZ,ZF5,* AP2ALPHA,ATF1,CEBPB,FOXM1,GCM,MZF1,SMAD3,SMAD4,TGIF,ZIC3
91	IMPA1	ENSG00000133731	-		*AP2REP,STAT5A,* AP3,NFY
91		ENSG00000104231	-	34967	*AP2REP,STAT5A,* AFP1,AML1,AREB6,EN1,GATA1,GKLF,IPF1,IRF1,MRF2,NFAT,NKX25,NKX62,OCT1,PAX2,PITX2,POU3F2,POU6F1,STAT,TATA,TST1
92		ENSG00000104231	-		*AML1,AREB6,GATA1,IPF1,MRF2,NFAT,OCT1,POU6F1,STAT5A,TST1,* AFP1,AP2REP,EN1,GKLF,IRF1,NKX25,NKX62,PAX2,PITX2,POU3F2,STAT,TATA
92		ENSG00000164695	+	11154	*AML1,AREB6,GATA1,IPF1,MRF2,NFAT,OCT1,POU6F1,STAT5A,TST1,* AP4,AR,CEBP,CEBPGAMMA,ELF1,FOXD3,GATA6,HNF3ALPHA,HOXA3,HOXA4,HSF2,MSX1,NFKAPPAB50,PEA3,POU1F1,S8,SMAD3,TITF1,ZIC2
93	EIF3S3	ENSG00000147677	-		*AREB6,STAT6,* AP2GAMMA,PEA3,PITX2
93		ENSG00000147679	+	10701	*AREB6,STAT6,* AFP1,AP2REP,CEBPDELTA,CEBPGAMMA,CREBP1CJUN,EVI1,FOXJ2,FXR,HMGIY,LHX3,LYF1,MRF2,MSX1,MYB,NKX22,OCT1,PU1,SRY,STAT3,TCF1P,TEF,VDR,YY1
94		ENSG00000147679	+		*LYF1,MRF2,* AFP1,AP2REP,AREB6,CEBPDELTA,CEBPGAMMA,CREBP1CJUN,EVI1,FOXJ2,FXR,HMGIY,LHX3,MSX1,MYB,NKX22,OCT1,PU1,SRY,STAT3,STAT6,TCF1P,TEF,VDR,YY1
94	RAD21	ENSG00000164754	-	108249	*LYF1,MRF2,* AML1,AP1,AP2ALPHA,ATF1,E2F1DP1,EN1,FOXD3,FOXM1,GATA1,GATA3,HOXA4,MAZR,NFAT,NKX25,SMAD3,SP1,SREBP1,STAT4,TBP
95	KIAA0020	ENSG00000080608	-		AP4,RSRFC4,STAT6,TEF1
95	RFX3	ENSG00000080298	-	681888	AFP1,AML1,AP1,AR,CEBP,CEBPA,CEBPB,CEBPDELTA,CEBPGAMMA,ERR1,EVI1,FOXD3,FREAC7,GATA1,GATA3,GCM,HOXA3,HSF1,IK1,LBP1,LHX3,LMO2COM,LYF1,MEIS1BHOXA9,MSX1,MYOGENIN,NFKB,OCT1,PEA3,S8,SMAD3,SOX5,STAT,STAT5B,TBP,TCF4
96	SPTAN1	ENSG00000119363	+		MAZ
96	WDR34	ENSG00000119333	-	104184	AP2REP,CEBP,FOXD3,NFMUE1,SP1,STAT5A,STAT5B,TCF4
97	SVIL	ENSG00000095756	-		*FOXM1,PAX4,* AP3,ATF6,CEBPA,FAC1,FOXD3,FOXJ2,HAND1E47,HSF2,IRF1,MYB,MYOGENIN,NKX25,PAX2,POU1F1,STAT3,STAT6,TEF,ZIC2
97		ENSG00000165757	-	473536	*FOXM1,PAX4,* COREBF,SMAD3,SRF,SRY,TEF1,USF2
98	H2AFY2	ENSG00000099284	+		*SRY,* AP1,AP2REP,CREL,DBP,HMGIY,HOXA3,HOXA4,LYF1,MYOD,NKX25,PAX2,PEA3,PU1,STAT6,TCF4,TEF1,USF2
98	AMID	ENSG00000042286	-	80089	*SRY,* AP2,AREB6,CEBPA,COUP,E12,GKLF,SP3,TBP
99	SARA1	ENSG00000079332	-		*AP2REP,PAX4,STAT1,* AP2GAMMA,CEBP,CEBPB,CEBPDELTA,EN1,ETF,GATA6,PEA3,STAT5A,TCF1P,ZIC3
99	PP	ENSG00000180817	-	62921	*AP2REP,PAX4,STAT1,* CREL,E12,ELF1,ERR1,GATA1,GATA3,HOXA4,LBP1,MTF1,NKX25,PBX1,PU1,SMAD3,SP1,STAT5B,TCF4
100	PSAP	ENSG00000148732	-		*PAX4,SF1,* STAT1,TCF11
100	CHST3	ENSG00000122863	+	113092	*PAX4,SF1,* AML1,AP1,AP2ALPHA,AP4,CACBP,CEBPA,CEBPB,CEBPGAMMA,CRX,ELK1,ETF,GATA1,GATA2,HOXA4,IRF1,LEF1,LYF1,MEIS1,MYOD,MZF1,NFE2,OCT1,P53,PAX2,PAX8,POU1F1,PU1,STAT,STAT5B,TFIII,ZID
101	ACTA2	ENSG00000107796	-		*AP2ALPHA,EN1,NKX25,OCT1,* AREB6,CACBP,CREBP1,DBP,FAC1,FOXD3,GATA1,IRF1,MEIS1,MMEF2,MRF2,MTF1,NFAT,POU1F1,STAT,STAT1,STAT5A,STAT5B,TFIII,ZIC1,ZIC3
101	TNFRSF6	ENSG00000026103	+	37770	*AP2ALPHA,EN1,NKX25,OCT1,* AML1,AP2REP,CDX2,CEBPGAMMA,E2F,HMGIY,HOXA4,MYB,MYOD,NFKAPPAB65,PAX2,PAX4,PEA3,RSRFC4,SP3,SRY,TCF4,TEF1,ZF5
102	TLL2	ENSG00000095587	-		*AP2REP,SF1,STAT5A,* AP2GAMMA,CEBPB,EN1,ERR1,GATA2,HSF1,IRF1,LFA1,MZF1,NFY,OSF2,P300,P53,PBX1,POU1F1,STAT,STAT5B,TAL1BETAITF2,TFIII,USF,ZIC1
102		ENSG00000077147	-	73134	*AP2REP,SF1,STAT5A,* AR,AREB6,CDC5,DBP,E12,E4F1,ETF,HNF3ALPHA,HOXA3,IPF1,LYF1,MSX1,NKX22,NKX25,PAX4,PEA3,PU1,SMAD3,STAT4,TITF1
103	ANKRD2	ENSG00000165887	+		*AP1,* AP2GAMMA,CACBP,PU1,STAT4

103	C10orf65	ENSG00000155252	+	11829	*AP1,* ALPHACP1,AP2ALPHA,ARP1,CDP,ELK1,EVI1,FOXM1,GATA3,GATA4,IRF1,LBP1,LFA1,LMO2COM,LYF1,MYB,MZF1,NFKAPPAB50,NFY,OCT1,PITX2,POU1F1,RORA1,SP1,TFIII,USF2,ZIC2
104	MGEA5	ENSG00000107835	-		SF1,SRY
104	KCNIP2	ENSG00000120049	-	25498	AP1,AP2,AREB6,CACBP,EVI1,GATA1,HEB,HMGIY,IK3,NKX22,NKX25,SMAD4,TGIF
105		ENSG00000177225	-		MZF1,SMAD4,TEL2,ZIC3
105	TALDO1	ENSG00000177156	+	2207	LFA1,MYOD,TCF4
106	LMO1	ENSG00000166407	-		*AP1,ISRE,LBP1,MEIS1,OCT1,* AML1,AP2,AP4,AREB6,CDP,CEBPB,COREBF,GATA2,HEB,HNF4ALPHA,IK1,MYOD,MZF1,NFKAPPAB,NFKB,NKX3A,NKX62,RP58,SMAD4,SOX5,SP1,SPZ1,SRY,STAT1,TCF1P,TFIII,USF,YY1,ZIC2
106	STK33	ENSG00000130413	-	316490	*AP1,ISRE,LBP1,MEIS1,OCT1,* AP2GAMMA,AP2REP,CDC5,CEBPGAMMA,ELF1,EN1,FREAC7,HOXA3,HOXA4,LYF1,NFY,P53,POU3F2,RSRFC4,SMAD3,SP3,STAT6,TCF4,TEF,TGIF,TITF1
107	API5	ENSG00000166181	+		ATF1,HIF1,LBP1,ZF5
107	TTC17	ENSG00000052841	+	46938	AP1,AREB6,ATF4,BACH2,CP2,E2F1,ELF1,EN1,EVI1,FOXD3,GATA1,GKLF,HSF1,IK2,IRF1,LEF1,MSX1,NFAT,PAX4,POU6F1,SRY,STAT4,TAL1BETAE47,TITF1,USF2
108	TP53I11	ENSG00000175274	-		*AREB6,EN1,SF1,ZIC2,* ALPHACP1,AP1,AP2ALPHA,CDP,CEBPA,CEBPB,COUP,GATA1,HAND1E47,HEB,HOXA4,MAZ,MYOGENIN,NFAT,PBX1,PEA3,SP1,SRY,STAT6,TCF1P,TCF4,TFIII,VDR
108	SYT13	ENSG00000019505	-	169576	*AREB6,EN1,SF1,ZIC2,* BRN2,CACBP,CDPCR3HD,DBP,ELF1,GATA4,HOXA3,LBP1,MEIS1,MEIS1AHOXA9,MSX1,MYB,MZF1,NKX25,PAX2,PITX2,POU1F1,PU1,STAT5A,STAT5B,TEF1,ZIC3
109	PANX1	ENSG00000110218	+		*AREB6,* AML1,AP2REP,AP4,CEBPB,LYF1,NKX3A,POU1F1,PU1,TGIF
109	GPR83	ENSG00000123901	-	272322	*AREB6,* DBP,GATA1,LMO2COM,MAZ,MEIS1BHOXA9,NFY,NKX25,NKX62,PAX2,PBX1,STAT5A,STAT6,TFIII
110	ROBO4	ENSG00000154133	-		*HMGIY,HOXA4,PU1,* AP2,BACH2,DBP,ETF,FOXO1,LMO2COM,MYOGENIN,NFKAPPAB,POU1F1,STAT,STAT3,STAT6,TCF4
110		ENSG00000165478	-	38205	*HMGIY,HOXA4,PU1,* AP1,AP2REP,CEBPB,CP2,E2F1,ELF1,FOXM1,GKLF,HFH4,LYF1,MAZ,MZF1,NFAT,NKX25,NKX62,P53,PEA3,STAT5A,TITF1,ZIC1,ZID
111	SCNN1A	ENSG00000111319	-		*AP2,AR,GATA3,HOXA4,OCT1,PU1,RREB1,SP1,* AP3,AP4,CDX2,DBP,E2F1DP1,EFC,EVI1,FOXM1,HNF3ALPHA,HOXA3,LBP1,MYOGENIN,MZF1,P300,P53,PEA3,TCF4,TEF1,TEL2,TFIII,TGIF,ZIC1,ZIC2
111	LTBR	ENSG00000111321	+	8607	*AP2,AR,GATA3,HOXA4,OCT1,PU1,RREB1,SP1,* AREB6,CETS1P54,CP2,E2F1DP2,ETF,HAND1E47,HEB,LFA1,LHX3,PAX2,SMAD3,SPZ1,SRY,STAT3,STAT5A,STAT5B,STAT6,USF2
112		ENSG00000165685	+		*STAT5B,* AP1FJ,AP3,CDC5,CDPCR3HD,CP2,EFC,ERR1,FOXD3,FOXM1,GATA1,GATA6,GKLF,MAZ,NKX3A,P300,PAX2,PBX1,SF1,SRY,STAT4,STAT5A
112	GABARAPL1	ENSG00000139112	+	33798	*STAT5B,* AP2REP,BACH2,CEBPGAMMA,LYF1,NKX25,PAX4,STAT6
113	FGD4	ENSG00000139132	+		AML1,AP2GAMMA,AP3,AP4,AREB6,CEBPB,CETS1P54,COREBF,CP2,DBP,FOXM1,HNF3ALPHA,HNF4ALPHA,LBP1,MTF1,OCT1,P53,SOX5,STAT5A,STAT5B,ZIC1
113	DNM1L	ENSG00000087470	+	177125	CDP,TGIF
114	DNM1L	ENSG00000087470	+		CDP,TGIF
114		ENSG00000139131	-	76615	HMGIY,HOXA4,MEIS1,POU1F1,SP1,TCF4,TEF
115		ENSG00000139131	-		*TCF4,* HMGIY,HOXA4,MEIS1,POU1F1,SP1,TEF
115	PKP2	ENSG00000057294	-	140856	*TCF4,* AFP1,AP1,AP3,AREB6,CACBP,GATA4,HEB,HSF2,NKX62,P53,PEA3,PU1,TBP
116	TUBA1	ENSG00000123416	-		ELF1,HEB,USF,ZIC2
116		ENSG00000139636	-	30643	AP2REP,CREBP1CJUN,GATA3,HMGIY,MYB,NKX22,SRY
117	SLC11A2	ENSG00000110911	-		*AREB6,NKX62,PITX2,TEF1,TGIF,* CEBPB,CEBPDELTA,EVI1,HSF2,IPF1,LYF1,MAZR,NFE2,PAX2,PU1,YY1
117	C12orf22	ENSG00000110925	-	31076	*AREB6,NKX62,PITX2,TEF1,TGIF,* AFP1,AML1,AP2ALPHA,AP3,AP4,AR,CDX2,CEBPA,CEBPGAMMA,CETS1P54,CP2,EFC,FAC1,FOXD3,FOXM1,FOXO4,GATA3,GCM,HAND1E47,HMGIY,HOXA4,ICSBP,LFA1,MMEF2,NFKAPPAB50,NKX22,NKX25,NKX3A,OCT1,PAX4,POU1F1,STAT,STAT5A,STAT5B,TFIII
118		ENSG00000177041	unknown		LFA1,TCF11,TFIII,ZIC3
118	MMP19	ENSG00000123342	-		AML1,AP2ALPHA,AP2REP,AP3,AP4,AREB6,CACBP,EN1,LYF1,MEIS1,MRF2,MSX1,NFKB,SF1,SMAD4,SP1
119	TMEM4	ENSG00000144785	-		*AP2REP,LFA1,* AP2,AREB6,E2F1,HSF2,ICSBP,IK1,MAZR,MSX1,NCX,OSF2,TCF4,ZF5
119	CS	ENSG00000062485	-	38732	*AP2REP,LFA1,* CDPCR3HD,CEBPB,DBP,E2F,GATA1,GATA4,IRF1,MZF1,OCT1,PAX2,PAX4,PEA3,PITX2,SF1,SP1,SRY,STAT5B,TEF1,YY1,ZIC2
120	SLC39A5	ENSG00000139540	+		*STAT3,* AP1,AP4,ATF3,ATF6,ETF,FOXO1,HOXA3,MYB,MZF1,NFAT,PEA3,PITX2,SF1,SP1,USF
120		ENSG00000139579	+	7992	*STAT3,* CP2,ERR1
121	TBC1D15	ENSG00000121749	+		*NKX25,SF1,* AP1,ICSBP,RORA1,STAT5A,TBP
121	TPH2	ENSG00000139287	+	99218	*NKX25,SF1,* AREB6,CDX2,CEBPB,CEBPGAMMA,CP2,ELF1,ERR1,FOXD3,FOXM1,FOXO4,GATA1,GATA6,HAND1E47,HMGIY,LYF1,NFAT,OCT1,PAX4,PBX1,TEF,TITF1
122		ENSG00000175504	unknown		*E2F1,MYOGENIN,PAX2,SP1,STAT6,* AML1,AP1,AP2ALPHA,AREB6,CEBPDELTA,DBP,FOXD3,FOXO4,GATA1,GATA4,HMGIY,HOXA4,LFA1,MSX1,NFAT,NKX25,PU1,SRY,STAT5A,TFIII
122	NUDT4	ENSG00000173598	+		*E2F1,MYOGENIN,PAX2,SP1,STAT6* , AP2,E47,EN1,GATA3,LYF1
123		ENSG00000136003	+		ATF3
123		ENSG00000176123	unknown		AP2GAMMA,AP2REP,DBP,ETF,GATA3,LFA1,LMO2COM,LYF1,MYOGENIN,POU1F1,SF1,SP1,STAT3
124	FBXW8	ENSG00000174989	+		NKX25,STAT5A,TCF1P,USF2,ZIC1
124		ENSG00000088992	-	188501	AP2ALPHA,CDC5,EN1,GATA2,MZF1,P53
125	KCTD4	ENSG00000180332	-		CDPCR3HD,EVI1,FOXD3,FOXM1,GATA1,NKX25,POU6F1,SOX5,STAT4,TCF4,TEF1
125	TPT1	ENSG00000133112	-	146445	AP2REP,ATF6,SP1,USF2
126	ITGBL1	ENSG00000139783	+		*NKX62,PAX2,POU1F1,SMAD3,STAT5B,* AFP1,AP1,AP3,EN1,HAND1E47,HOXA3,IK3,IPF1,LYF1,MZF1,PBX1,PU1,TBP,TCF1P
126	FGF14	ENSG00000102466	-	464034	*NKX62,PAX2,POU1F1,SMAD3,STAT5B,* AML1,AP4,AR,CETS1P54,CHOP,CREBP1CJUN,CRX,E2F1,E47,EVI1,GATA1,HSF2,ICSBP,IK1,LBP1,LFA1,MSX1,NFE2,NFY,NKX22,NKX25,OCT1,P53,STAT6,TCF11,TCF4,TFIII
127	CFL2	ENSG00000165410	-		LYF1,MAZ,PITX2
127	BAZ1A	ENSG00000092277	-	160929	AP1,AP2REP,AREB6,CEBPDELTA,CREBP1CJUN,ELK1,EVI1,FOXM1,GATA6,HNF3ALPHA,ISRE,LEF1,LHX3,MEIS1,MRF2,NFE2,NFY,NKX22,NKX25,NKX3A,OCT1,PBX1,POU1F1,SF1,SRY,STAT1,STAT5A,STAT5B,STAT6,TEF,TFIIA,ZF5,ZIC1
128	KIAA1596	ENSG00000092191	+		*OCT1,* PBX1,SRY,TBP
128	C14orf106	ENSG00000129534	-	110991	*OCT1,* AREB6,CDPCR3HD,DBP,EN1,FOXD3,IRF1,NKX3A,PAX4,TGIF
129		ENSG00000170348	-		*AML1,AP2REP,EN1,LYF1,* AP1FJ,BRN2,CACBP,CDC5,CDPCR3HD,CEBPGAMMA,COUP,ETF,FOXM1,FREAC3,HFH4,HNF4ALPHA,P53,PAX2,SF1,SRY,STAT,TATA,TCF4,TFIII
129	FOS	ENSG00000170345	+	102193	*AML1,AP2REP,EN1,LYF1,* AP1,AP4,AREB6,ATF1,ELF1,EVI1,FOXO1,FOXO4,GATA1,HFH3,HNF3ALPHA,IRF1,LBP1,LFA1,LMO2COM,MAZ,MRF2,MYB,MZF1,NFAT,NFE2,OCT1,PEA3,POU1F1,RORA1,RREB1,S8,SMAD4,SOX5,SP3,STAT1,STAT3,STAT4,STAT5A,TBP,TCF11,TGIF,TST1,YY1,ZIC2
130	C14orf4	ENSG00000119669	-		*AP4,AREB6,ATF3,FOXM1,GATA3,HNF3ALPHA,IRF1,SRF,SRY,STAT3,* AHRARNT,AML1,AP2,CACBP,CEBPA,CETS1P54,COUP,CREBP1,E2F1,E47,EN1,FOXO4,GATA1,GATA6,GCM,HNF6,HOXA4,HSF1,HSF2,LBP1,LEF1,LYF1,MTF1,MZF1,NFKAPPAB65,NFMUE1,NKX62,NRF1,OCT1,OSF2,P300,PAX2,PAX4,PBX1,PEA3,PITX2,POU1F1,POU6F1,PU1,SP1,SPZ1,TCF1P,TITF1,ZIC3
130	KIAA1737	ENSG00000133965	+	70526	*AP4,AREB6,ATF3,FOXM1,GATA3,HNF3ALPHA,IRF1,SRF,SRY,STAT3,* ARNT,ATF1,CEBPB,E2F1DP1,FOXJ2,GATA2,HIF1,HMGIY,HNF4ALPHA,LHX3,LMO2COM,MRF2,MYB,NKX22,NKX61,STAT5A,STAT5B,TBP,TEF1,ZIC1
131	AK7	ENSG00000140057	+		STAT5A
131	PAPOLA	ENSG00000090060	+	110349	AFP1,AP3,CEBPB,CHOP,EN1,HOXA4,HSF1,LHX3,LYF1,MYB,PU1,SF1,SRY
132	ZFP106	ENSG00000103994	-		*GATA1,* CEBPB,E47,EN1,EVI1,FAC1,GATA6,HMGIY,HNF3ALPHA,IK1,IRF1,MEIS1,NKX25,NKX61,OCT1,PBX1,PU1,SF1,SOX5,SP1,TCF1P,TST1,ZID
132	SNAP23	ENSG00000092531	+	38070	*GATA1,* CEBPGAMMA,FOXD3,IK2,TCF11
133	SNAP23	ENSG00000092531	+		CEBPGAMMA,FOXD3,GATA1,IK2,TCF11
133		ENSG00000180979	-	53165	AP1,AP2,AP2GAMMA,AP2REP,AP3,CDC5,CDP,CEBP,CEBPDELTA,ETF,FAC1,FOXM1,GCM,IRF1,MAZ,NKX61,OCT1,P53,PAX4,PBX1,POU1F1,STAT,TEF1,TITF1,ZIC2,ZID
134	MPI	ENSG00000178802	+		*STAT6,* CETS1P54,EN1,STAT1,ZF5
134	C15orf17	ENSG00000178761	-	15979	*STAT6,* AML1,AP1,MYB,SRY,TEF1
135	NDUFB10	ENSG00000140990	+		FOXO1,GATA1,GATA6,LBP1,MEIS1BHOXA9,TBP
135	RPS2	ENSG00000140988	-	5300	AP2REP,CP2,GCM,SRY
136	TFAP4	ENSG00000090447	-		*AP4,STAT6,* AP2GAMMA,AREB6,ATF1,CEBP,CEBPA,CETS1P54,CREB,DBP,E2F1DP2,EN1,FAC1,FOXO4,GKLF,IK1,LYF1,MYCMAX,MYOD,OCT1,PEA3,PITX2,POU1F1,SP1,SRY,STAT5A,TAXCREB,TCF4,TEF1
136	GLIS2	ENSG00000126603	+	59250	*AP4,STAT6,* AP2ALPHA,E12,FOXD3,IRF1,MMEF2,PAX2,SF1,SPZ1,TFIII,TGIF
137	GLIS2	ENSG00000126603	+		*SF1,* AP2ALPHA,AP4,E12,FOXD3,IRF1,MMEF2,PAX2,SPZ1,STAT6,TFIII,TGIF
137		ENSG00000103426	-	84703	*SF1,* AP2GAMMA,AP3,CACBP,DBP,HIF1,LBP1,NRF1,P300,STAT3,STAT5B,TBP
138		ENSG00000149930	+		*BACH2,IK2,LYF1,MZF1,SRY,* AML1,AP1FJ,AP2GAMMA,AP4,AREB6,CDPCR3HD,CP2,CRX,E2F1DP1,HAND1E47,HSF2,LBP1,MYOD,OSF2,PU1,STAT4,TFIII,USF
138	HIRIP3	ENSG00000149929	-	22170	*BACH2,IK2,LYF1,MZF1,SRY,* AP2,ATF6,CEBPGAMMA,HIF1,LFA1,MAZ,MAZR,MEIS1,NFMUE1,P300,PAX4,SF1,SP1,STAT5A,TCF11,ZF5,ZIC3
139	VPS35	ENSG00000069329	-		*GATA1,MYB,SOX5,STAT5A,TEF1,* CEBPGAMMA,EN1,GATA2,MZF1,STAT5B,TATA
139	ORC6L	ENSG00000091651	+	467	*GATA1,MYB,SOX5,STAT5A,TEF1,* AP3,E2F1,HOXA3,NFY,OCT1,SMAD3,SP1,SREBP1,STAT4,TFIIA
140	ORC6L	ENSG00000091651	+		*GATA1,* AP3,E2F1,HOXA3,MYB,NFY,OCT1,SMAD3,SOX5,SP1,SREBP1,STAT4,STAT5A,TEF1,TFIIA
140		ENSG00000140795	-	58657	*GATA1,* GKLF,HFH4,HNF3ALPHA,MAZ,MZF1,TCF1P,TFIII,TST1
141		ENSG00000006740	+		*P300,* AP2REP,CDC5,CDP,CREB,E2F,ELF1,HLF,HNF3ALPHA,LHX3,LYF1,MEIS1,MYB,NKX62,OCT1,SF1,SP3,SREBP1,STAT,STAT4,TCF1P,TGIF,USF2
141	ELAC2	ENSG00000006744	-	228482	*P300,* AP4,CDX2,PAX2,POU1F1,RP58,STAT5A
142	ELAC2	ENSG00000006744	-		*PAX2,STAT5A,* AP4,CDX2,P300,POU1F1,RP58
142	HS3ST3A1	ENSG00000153976	-	583906	*PAX2,STAT5A,* AREB6,ATF1,ATF3,CDP,CEBPA,CETS1P54,COREBF,CRX,DBP,ETF,FOXO1,HNF3ALPHA,HOXA3,IPF1,LMO2COM,MEIS1,MYB,MYOD,MYOGENIN,NFAT,OCT1,P53,PAX4,PAX8,PBX1,POU6F1,SP3,STAT5B,TAXCREB,TEF,TITF1,VDR,ZF5,ZIC2
143	WSB1	ENSG00000109046	+		*HNF3ALPHA,SRY,TCF4,* CDX2,FOXD3,LBP1,OSF2,STAT5A,TBP
143	KSR	ENSG00000141068	+	177953	*HNF3ALPHA,SRY,TCF4,* AML1,AP2ALPHA,AP2GAMMA,AREB6,ATF4,CDPCR3HD,DBP,FOXO1,GATA1,GATA3,GKLF,LEF1,LFA1,MAZ,MAZR,MYOGENIN,MZF1,NFKAPPAB50,NFMUE1,PEA3,POU1F1,PU1,SMAD3,SMAD4,SOX5,SP1,SREBP1,STAT4,STAT5B,TEF1,ZIC2,ZID
144	RPL23A	ENSG00000173070	+		*AP2ALPHA,CACBP,USF2,* ELK1,GATA6,P53,PEA3,SP1,STAT6
144		ENSG00000160606	-	6230	*AP2ALPHA,CACBP,USF2,* AML1,AP1,AP1FJ,AP4,AREB6,CEBPGAMMA,E2F1,ETF,HNF1,IRF1,LBP1,MEIS1,MYB,MZF1,NFKAPPAB,NFY,STAT,TCF1P,TFIII,VDR
145		ENSG00000108256	-		AP2,AP2REP,AREB6,CEBPB,DBP,EN1,FOXM1,HLF,LHX3,LMO2COM,LYF1,MRF2,NKX25,NKX61,NKX62,PAX4,POU3F2,SRY,STAT1,STAT5A,STAT5B,STAT6,TST1
145		ENSG00000160551	+	157220	IK2,MYOGENIN,TCF11
146		ENSG00000141302	+		AP3,BACH2,CDPCR3HD,CEBPB,CHOP,CREL,CRX,E2F,ERR1,FOXD3,FOXM1,FOXO1,GKLF,HAND1E47,HNF4ALPHA,ICSBP,IPF1,IRF1,LYF1,MYB,NCX,NFKAPPAB50,NKX22,NKX25,NKX61,OCT1,PEA3,SP3,SRY,STAT1,STAT6,TBP,TFIII,VDR,ZIC1,ZIC3
146		ENSG00000167549	-	29155	
147		ENSG00000172653	-		*DBP,* AP2ALPHA,AP4,CACBP,EVI1,HNF4ALPHA,IK2,LFA1,OSF2,SRY,STAT5B
147	CCL5	ENSG00000161570	-	11486	*DBP,* ARNT,GATA1,HLF,IK1,LMO2COM,NFKAPPAB65,PAX2,STAT6,TATA,TEF1
148	DDX52	ENSG00000141141	-		*GATA1,MZF1,SRY,STAT5B,STAT6,* AP1,AP2REP,CEBP,CHX10,FOXO1,FOXO3,HNF6,HOXA3,HOXA4,LFA1,MAZ,MYCMAX,NCX,NKX25,P300,PAX2,PAX4,STAT4,STAT5A,TCF11,TCF4,TEF1
148	TCF2	ENSG00000108753	-	101584	*GATA1,MZF1,SRY,STAT5B,STAT6* , AP2,COUP,HLF,IRF1,OCT1,STAT3
149		ENSG00000167131	+		*CACCCBF,LMO2COM,STAT5A,STAT6* , CACBP,E2F1,FOXO1,MAZ,NFAT,NFKB,P300,PU1,RORA1,SF1
149	GFAP	ENSG00000131095	-	15726	*CACCCBF,LMO2COM,STAT5A,STAT6,* AP2ALPHA,AP2GAMMA,AP4,BACH1,GATA1,GKLF,HOXA3,IK2,LYF1,MEIS1,MZF1,P53,PAX2,SP3,STAT3,TFIII,ZIC1
150	SGCA	ENSG00000108823	+		*AML1,AP2REP,AP4,AREB6,MAZR,NFAT,OCT1,OSF2,POU1F1,TCF11,TGIF,ZF5,* AP1,AP2,AP2GAMMA,AP3,CEBP,CRX,ELF1,FOXD3,GATA1,GKLF,HMGIY,LFA1,LYF1,MYOGENIN,NCX,PU1,RSRFC4,SF1,SMAD3,SRY,TST1,USF2
150	COL1A1	ENSG00000108821	-	35627	*AML1,AP2REP,AP4,AREB6,MAZR,NFAT,OCT1,OSF2,POU1F1,TCF11,TGIF,ZF5,* ALPHACP1,AP1FJ,ATF1,ATF4,BACH2,CACBP,CEBPB,COREBF,E2F1,E2F1DP1,ERR1,GATA4,GCM,HAND1E47,IK1,IK3,IRF1,LBP1,MEIS1,NKX25,NMYC,P53,PAX4,RREB1,TAL1BETAE47,TCF4,TFIIA,TFIII,TITF1
151		ENSG00000141576	-		*AREB6,HNF4ALPHA,* AFP1,AML1,AP1,AP3,CDX2,CEBP,ETF,FOXJ2,FOXO1,FOXO4,GKLF,HMGIY,HOXA4,LYF1,MAZ,MEIS1,MZF1,NFE2,NKX61,PAX2,PEA3,PITX2,POU6F1,SF1,STAT6,TCF1P,ZIC1

151		ENSG00000129646	-	67371	*AREB6,HNF4ALPHA,* AP2ALPHA,ARNT,CEBPB,CP2,EN1,FAC1,GATA4,GATA6,GCM,MYCMAX,OCT1,OSF2,PU1,USF2
152	TBC1D16	ENSG00000167291	-		*CEBPDELTA,* AP1,DBP,ETF,STAT5B,USF,ZIC1
152		ENSG00000141519	+	815	*CEBPDELTA,* AML1,AP2REP,AP3,E12,E2F1DP1RB,FAC1,HEB,HOXA3,IK2,IRF1,MAZ,MEIS1,MYB,MYOD,OSF2,PBX1,SF1,SMAD4,SRY,STAT3,TCF4
153	THOC1	ENSG00000079134	-		*HNF3ALPHA,MZF1,* AP2REP,AP4,CDPCR3HD,CP2,E2F1,EVI1,FOXJ2,MYB,NFAT,OCT1,P53,SF1,SMAD3,SP3,TITF1,USF
153	COLEC12	ENSG00000158270	-	232681	*HNF3ALPHA,MZF1,* CDC5,CEBPB,ELF1,FOXM1,FOXO1,HEB,HOXA4,IRF1,LBP1,LMO2COM,LYF1,NFY,PAX4,PU1,SRY,STAT3,STAT6,ZF5,ZID
154	C18orf9	ENSG00000101624	-		*LYF1,* YY1,ZIC1
154	PTPN2	ENSG00000175354	-	181636	*LYF1,* AML1,AP3,CEBPB,CRX,EN1,HIF1,HOXA3,LBP1,LEF1,MSX1,NFAT,NRF1,OSF2,PAX8,SP1,TFIIA
155	MAPRE2	ENSG00000166974	+		*POU1F1,* AP2GAMMA,AREB6,CACBP,CEBPB,ELK1,EVI1,FOXO4,GATA1,GATA2,GATA4,HEB,HNF3ALPHA,IRF1,LEF1,LFA1,LYF1,MAZ,MYOD,MZF1,NFY,OCT1,P53,PAX2,PAX4,POU6F1,SMAD3,SRY,STAT,STAT1,STAT6,TAL1ALPHAE47,TITF1,YY1,ZIC3
155		ENSG00000118267	+	199634	*POU1F1,* FOXD3,TCF11
156	SLC14A2	ENSG00000132874	+		*AP1,AP2REP,CACBP,CP2,FOXD3,HAND1E47,LFA1,LYF1,NFY,OCT1,STAT,STAT6,USF2,* AP2ALPHA,CDX2,E2F,ERR1,EVI1,FOXO1,FREAC7,HEB,HMEF2,IK3,LHX3,MZF1,P300,PEA3,SREBP1,SRY,TCF1P,TFIII,YY1
156	SLC14A1	ENSG00000141469	+	99563	*AP1,AP2REP,CACBP,CP2,FOXD3,HAND1E47,LFA1,LYF1,NFY,OCT1,STAT,STAT6,USF2,* AP3,AP4,AREB6,CEBPGAMMA,CETS1P54,CREB,CRX,DBP,HNF3ALPHA,HNF6,MTF1,MYB,MYOGENIN,NCX,NKX25,NKX62,RORA1,SF1,STAT5B,TST1,VDR
157	OAZ1	ENSG00000104904	+		ELF1
157	LSM7	ENSG00000130332	-	59051	AP2ALPHA,AP2REP,DBP,ETF,FOXM1,HAND1E47,HMGIY,PAX2,SRY,STAT5A,STAT5B,ZIC3
158	SAFB2	ENSG00000130254	-		*ELF1,SP1,* AP3,CEBP,DBP,E2F1,NRF1,PBX1,PU1,TCF4,YY1
158	SAFB	ENSG00000160633	+	362	*ELF1,SP1,* AP1,CETS1P54,CHOP,EN1,HOXA4,LYF1,MAZ,NFMUE1,PAX3,SRY,STAT3,STAT5A,TITF1
159	HOOK2	ENSG00000095066	-		*AML1,AP1,DBP,E2F1,ELK1,EVI1,FOXD3,FREAC4,GATA1,GCM,HMGIY,IK1,IK3,LMO2COM,MAZR,MEIS1,MMEF2,NFKAPPAB,NFMUE1,NKX22,OCT1,PAX2,RORA1,SRF,TAL1BETAE47,YY1,ZIC2,* AR,COREBF,FAC1,FOXO4,GATA3,GKLF,HAND1E47,HEB,IRF1,MTF1,NKX25,NKX62,NMYC,POU1F1,SPZ1,STAT,TBP,TCF11,TCF4,TEL2,ZF5
159	JUNB	ENSG00000171223	+	15953	*AML1,AP1,DBP,E2F1,ELK1,EVI1,FOXD3,FREAC4,GATA1,GCM,HMGIY,IK1,IK3,LMO2COM,MAZR,MEIS1,MMEF2,NFKAPPAB,NFMUE1,NKX22,OCT1,PAX2,RORA1,SRF,TAL1BETAE47,YY1,ZIC2,* CETS1P54,E2F1DP1,EFC,HFH8,HNF6,IK2,PEA3,SP1,STAT1,STAT5A,STAT5B,USF2,ZIC3
160	CHERP	ENSG00000085872	-		*GATA1,STAT5A,* E2F1,LBP1,LMO2COM,MZF1,PAX4,SRY,TEL2,USF,ZIC1
160	SLC35E1	ENSG00000127526	-	29745	*GATA1,STAT5A,* AML1,CETS1P54,ERR1,PEA3,SF1,TBP
161	UBB	ENSG00000006717	+		*ZIC1,* CETS1P54,CREB,E2F4DP2,ETF,GATA1,MSX1,NRF1,PU1
161		ENSG00000006015	+	16938	*ZIC1,*
162	HAMP	ENSG00000105697	+		AP2REP,AREB6,LMO2COM,STAT
162	MAG	ENSG00000105695	+	9623	ZIC1
163	GYS1	ENSG00000104812	-		*TGIF,* PU1,ZIC3
163	CGB2	ENSG00000104818	+	382	*TGIF,* PITX2
164	DPM1	ENSG00000000419	-		AP2ALPHA,AP2REP,ATF1,ATF6,CEBPB,E2F1,ETF,LYF1,MSX1,PEA3,STAT4,TEF1
164	MOCS3	ENSG00000124217	+	276	FREAC7,HNF3ALPHA,IPF1,LEF1,LMO2COM,NCX,NFAT,NFY,OCT1,P53,PAX4,PBX1,SMAD3
165	POLDIP3	ENSG00000100227	-		STAT5A
165	DIA1	ENSG00000100243	-	32121	FOXM1,PAX2
166	HDAC10	ENSG00000100429	-		*AP2GAMMA,COREBF,MZF1,PEA3,* AP2REP,MAZ,MAZR,OSF2,STAT5A,ZF5
166		ENSG00000073196	unknown		*AP2GAMMA,COREBF,MZF1,PEA3,* AP1,CDPCR3HD,CETS1P54,E2F1,E47,GKLF,LYF1,MYOD,NRF1,SP1
167		ENSG00000157600	+		*CHOP,PAX4,TFIII,* AP3,GATA4,GKLF,LFA1,MYB,P300,SMAD3,SP1,SRF
167	AMMECR1	ENSG00000101935	-	315129	*CHOP,PAX4,TFIII,* AP2ALPHA,AP4,BRN2,CDX2,CEBPGAMMA,EVI1,FOXM1,FOXO4,HNF1,HNF3ALPHA,LYF1,MSX1,OCT1,POU3F2,PU1,RREB1,STAT5A,TST1,ZIC3
168	FHL1	ENSG00000022267	+		*DBP,MAZ,STAT5A,ZIC3,* AML1,AP1FJ,CACBP,GATA2,HFH4,MZF1,PAX2,TCF1P,TFIII,TITF1
168		ENSG00000129680	-	103852	*DBP,MAZ,STAT5A,ZIC3,* AREB6,CETS1P54,HMGIY,HOXA4,IRF1,NFE2,NKX25,OCT1,PAX4,POU1F1,SMAD4,SP1,SRY,STAT,STAT3,STAT6,USF2
169	MTMR1	ENSG00000063601	+		*AP2ALPHA,DBP,* MYOGENIN,PAX4,TGIF
169	CD99L2	ENSG00000102181	-	205234	*AP2ALPHA,DBP,* AP1,AP2GAMMA,AP2REP,AREB6,CDC5,CEBPGAMMA,COREBF,CRX,ERR1,FOXO4,FREAC4,HSF1,MAZ,MRF2,NKX25,OCT1,PAX2,POU6F1,SRY,TCF4
170	ATP6AP1	ENSG00000071553	+		AP2ALPHA,AP2REP,E2F1,E2F1DP1,FOXM1,MSX1
170	GDI1	ENSG00000102129	+	8456	CEBPB,ETF,TEF1,YY1,ZF5
171	FUNDC2	ENSG00000165775	+		*PAX4,*
171		ENSG00000165778	unknown		*PAX4,* AP2GAMMA,AREB6,BACH1,BRN2,CDX2,CEBP,CEBPB,E2F,E2F1DP1,ELK1,EN1,EVI1,FOXO3,FOXO4,GATA1,HFH4,HMGIY,HNF3ALPHA,HNF6,IK2,IPF1,LEF1,LFA1,LYF1,MAZ,MSX1,MYOD,MZF1,NFE2,NKX62,OCT1,OSF2,PEA3,SF1,STAT3,STAT5B,TCF11,TEF,TFIII,ZF5
