# Supplementary material for: Chromosomal clustering of a human transcriptome reveals regulatory background
Source: BMC Bioinformatics. 2005 Sep 19;6:230. doi: 10.1186/1471-2105-6-230 (PMC1261156; doi:10.1186/1471-2105-6-230)
Supplement: Additional File 2 — Gene Ontology terms shared by gene clusters. Shown are clustered heart-expressed genes annotated with Ensembl gene IDs, HUGO gene names and shared Gene Ontology (GO) terms of layer 4 and 5. The GO term IDs and descriptions are given. [file 1471-2105-6-230-S2.pdf]

Table 2S – Gene Ontology terms shared by gene clusters

1st Ensembl-Gene	2nd Ensembl-Gene	1st Hugo-Name	2nd Hugo-Name	Common GO-Terms in Layer 4	Common GO-Terms in Layer 5
**ENSG00000008125**	**ENSG00000008128**	**MIFR-2**	**CDC2L1**	GO:0019538	
**ENSG00000175279**	**ENSG00000160049**	**CORT**	**DFFA**	GO:0003677,GO:0007242	
**ENSG00000153898**	**ENSG00000055732**	**MCOLN2**	**MCOLN3**	GO:0015268,GO:0006811	GO:0005216,GO:0006812
**ENSG00000162654**	**ENSG00000154451**	**GBP4**	**GBP5**	GO:0017076,GO:0016817,GO:0009607	GO:0019001,GO:0016818,GO:0006952
**ENSG00000084754**	**ENSG00000138029**	**HADHA**	**HADHB**	GO:0016614,GO:0016746,GO:0016835,GO:0005737,GO:0006629,GO:0006082	GO:0016616,GO:0016747,GO:0016836,GO:0005739,GO:0006631,GO:0019752
**ENSG00000143947**	**ENSG00000085760**	**RPS27A**	**MTIF2**	GO:0005737,GO:0009058,GO:0019538	GO:0009059,GO:0006412
**ENSG00000144021**	**ENSG00000144028**	unknown	**ASCC3L1**	GO:0005634,GO:0006139	
**ENSG00000088179**	**ENSG00000115109**	**PTPN4**	**EPB41L5**	GO:0005737	GO:0005856
**ENSG00000138443**	**ENSG00000173166**	**ABI2**	**RAPH1**	GO:0005737	
**ENSG00000173166**	**ENSG00000178562**	**RAPH1**	**CD28**	GO:0007166	
**ENSG00000178562**	**ENSG00000163599**	**CD28**	**CTLA4**	GO:0005887,GO:0016021,GO:0009607	GO:0005887,GO:0006952
**ENSG00000134086**	**ENSG00000134070**	**VHL**	**IRAK2**	GO:0019538	
**ENSG00000131368**	**ENSG00000131381**	**MRPS25**	**ZFYVE20**	GO:0005737	
**ENSG00000145284**	**ENSG00000138674**	**SCD4**	**SEC31L1**	GO:0005737	
**ENSG00000052802**	**ENSG00000109472**	**SC4MOL**	**CPE**	GO:0005886	
**ENSG00000132356**	**ENSG00000145592**	**PRKAA1**	**RPL37**	GO:0009058,GO:0019538	GO:0009059
**ENSG00000113318**	**ENSG00000113319**	**MSH3**	**RASGRF2**	GO:0017076	
**ENSG00000152700**	**ENSG00000113615**	**SARA2**	**SEC24A**	GO:0005737,GO:0015031	GO:0005783,GO:0005794,GO:0006886
**ENSG00000177895**	**ENSG00000177839**	**PCDHB16**	**PCDHB9**	GO:0016337,GO:0019226,GO:0009887,GO:0016021	GO:0007156,GO:0007268,GO:0007399
**ENSG00000122566**	**ENSG00000122565**	**HNRPA2B1**	**CBX3**	GO:0005634,GO:0006139	
**ENSG00000167031**	**ENSG00000147454**	**LYSAL1**	unknown	GO:0005737	
**ENSG00000136933**	**ENSG00000044574**	unknown	**HSPA5**	GO:0005737	
**ENSG00000156510**	**ENSG00000156515**	unknown	**HK1**	GO:0016772,GO:0019200,GO:0017076,GO:0005975,GO:0006066,GO:0009056,GO:0006091	GO:0016773,GO:0004396,GO:0030554,GO:0006096,GO:0015980
**ENSG00000079332**	**ENSG00000180817**	**SARA1**	**PP**	GO:0016817	GO:0016818
**ENSG00000148732**	**ENSG00000122863**	**PSAP**	**CHST3**	GO:0005737,GO:0016021	
**ENSG00000107796**	**ENSG00000026103**	**ACTA2**	**TNFRSF6**	GO:0005737	
**ENSG00000119912**	**ENSG00000138160**	**IDE**	**KIF11**	GO:0005737	
**ENSG00000110218**	**ENSG00000123901**	**PANX1**	**GPR83**	GO:0016021	
**ENSG00000111319**	**ENSG00000111321**	**SCNN1A**	**LTBR**	GO:0016021	
**ENSG00000069493**	**ENSG00000110848**	unknown	**CD69**	GO:0004888,GO:0005529,GO:0005887,GO:0016021,GO:0009607,GO:0016337,GO:0007166	GO:0005887,GO:0006952,GO:0007157
**ENSG00000087470**	**ENSG00000139131**	**DNM1L**	unknown	GO:0017076	
**ENSG00000102738**	**ENSG00000102743**	**MRPS31**	**SLC25A15**	GO:0005737	GO:0005739
**ENSG00000068650**	**ENSG00000126217**	**ATP11A**	**MCF2L**	GO:0017076	
**ENSG00000165410**	**ENSG00000092277**	**CFL2**	**BAZ1A**	GO:0005634,GO:0005737	GO:0005856
**ENSG00000140990**	**ENSG00000140988**	**NDUFB10**	**RPS2**	GO:0005737	
**ENSG00000008516**	**ENSG00000008517**	**MMP25**	unknown	GO:0009607	GO:0006952
**ENSG00000109046**	**ENSG00000141068**	**WSB1**	**KSR**	GO:0007242	
**ENSG00000108823**	**ENSG00000108821**	**SGCA**	**COL1A1**	GO:0009887	
**ENSG00000132874**	**ENSG00000141469**	**SLC14A2**	**SLC14A1**	GO:0016021,GO:0015204,GO:0015268,GO:0015837	GO:0015204,GO:0015840
**ENSG00000152229**	**ENSG00000152234**	**PSTPIP2**	**ATP5A1**	GO:0005737	
**ENSG00000130254**	**ENSG00000160633**	**SAFB2**	**SAFB**	GO:0003677,GO:0005634	
**ENSG00000090659**	**ENSG00000104938**	**CD209**	**CD209L**	GO:0005529	
**ENSG00000125753**	**ENSG00000125741**	**VASP**	**OPA3**	GO:0005737	GO:0005856
**ENSG00000142541**	**ENSG00000142534**	**RPL13A**	**RPS11**	GO:0005737,GO:0030529,GO:0009058,GO:0019538	GO:0005840,GO:0009059,GO:0006412
**ENSG00000123872**	**ENSG00000175885**	unknown	**ZNF611**	GO:0005634,GO:0006139	GO:0006350
**ENSG00000170954**	**ENSG00000170949**	**ZNF415**	**ZNF160**	GO:0006139	GO:0006350
**ENSG00000000419**	**ENSG00000124217**	**DPM1**	**MOCS3**	GO:0009058	
**ENSG00000142192**	**ENSG00000166265**	**APP**	**CYYR1**	GO:0016021	
**ENSG00000159113**	**ENSG00000142166**	**IL10RB**	**IFNAR1**	GO:0004888,GO:0019955,GO:0016021,GO:0009607	GO:0004896
**ENSG00000160216**	**ENSG00000160218**	**AGPAT3**	**TMEM1**	GO:0016021	
**ENSG00000100413**	**ENSG00000172346**	**POLR3H**	unknown	GO:0006139	GO:0006350

GO-Term layer 4	Description
GO:0003677	DNA binding
GO:0004888	transmembrane receptor activity
GO:0005529	sugar binding
GO:0005634	nucleus
GO:0005737	cytoplasm
GO:0005886	plasma membrane
GO:0005887	integral to plasma membrane
GO:0005975	carbohydrate metabolism
GO:0006066	alcohol metabolism
GO:0006082	organic acid metabolism
GO:0006091	generation of precursor metabolites and energy
GO:0006139	nucleobase, nucleoside, nucleotide and nucleic acid metabolism
GO:0006629	lipid metabolism
GO:0006811	ion transport
GO:0007166	cell surface receptor linked signal transduction
GO:0007242	intracellular signaling cascade
GO:0009056	catabolism
GO:0009058	biosynthesis
GO:0009607	response to biotic stimulus
GO:0009887	organogenesis
GO:0015031	protein transport
GO:0015204	urea transporter activity
GO:0015268	alpha-type channel activity
GO:0015837	amine transport
GO:0016021	integral to membrane
GO:0016337	cell-cell adhesion
GO:0016614	oxidoreductase activity, acting on CH-OH group of donors
GO:0016746	transferase activity, transferring acyl groups
GO:0016772	transferase activity, transferring phosphorus-containing groups
GO:0016817	hydrolase activity, acting on acid anhydrides
GO:0016835	carbon-oxygen lyase activity
GO:0017076	purine nucleotide binding
GO:0019200	carbohydrate kinase activity
GO:0019226	transmission of nerve impulse
GO:0019538	protein metabolism
GO:0019955	cytokine binding
GO:0030529	ribonucleoprotein complex

GO-Term layer 5	Description
GO:0004396	hexokinase activity
GO:0004896	hematopoietin/interferon-class (D200-domain) cytokine receptor activity
GO:0005216	ion channel activity
GO:0005739	mitochondrion
GO:0005783	endoplasmic reticulum
GO:0005794	Golgi apparatus
GO:0005840	ribosome
GO:0005856	cytoskeleton
GO:0005887	integral to plasma membrane
GO:0006096	glycolysis
GO:0006350	transcription
GO:0006412	protein biosynthesis
GO:0006631	fatty acid metabolism
GO:0006812	cation transport
GO:0006886	intracellular protein transport
GO:0006952	defense response
GO:0007156	homophilic cell adhesion
GO:0007157	heterophilic cell adhesion
GO:0007268	synaptic transmission
GO:0007399	neurogenesis
GO:0009059	macromolecule biosynthesis
GO:0015204	urea transporter activity
GO:0015840	urea transport
GO:0015980	energy derivation by oxidation of organic compounds
GO:0016616	oxidoreductase activity, acting on the CH-OH group of donors, NAD or NADP as ac
GO:0016747	transferase activity, transferring groups other than amino-acyl groups
GO:0016773	phosphotransferase activity, alcohol group as acceptor
GO:0016818	hydrolase activity, acting on acid anhydrides, in phosphorus-containing anhydride
GO:0016836	hydro-lyase activity
GO:0019001	guanyl nucleotide binding
GO:0019752	carboxylic acid metabolism
GO:0030554	adenyl nucleotide binding
