# Supplementary figures and images for: Chromosomal clustering of a human transcriptome reveals regulatory background
Source: BMC Bioinformatics. 2005 Sep 19;6:230. doi: 10.1186/1471-2105-6-230 (PMC1261156; doi:10.1186/1471-2105-6-230)

Figure 1S

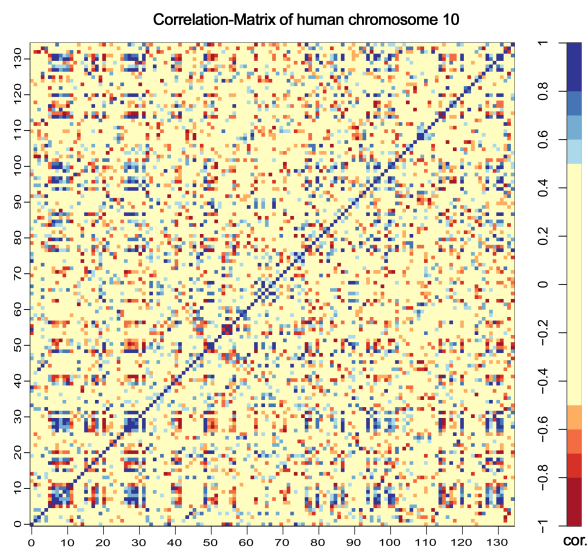

Supplement: Additional File 3 — Correlation matrix of human chromosome 10. The matrix of Pearson correlation coefficients between the expression profiles of heart-expressed genes is shown color-coded, with genes being arranged according to their order on the chromosome. Gene pairs with similar expression levels are depicted in blue, anti-correlated pairs are shown in red. [file 1471-2105-6-230-S3.pdf]
